# Towards a universal model of family centered care: a scoping review

**DOI:** 10.1186/s12913-019-4394-5

**Published:** 2019-08-13

**Authors:** Kristina M. Kokorelias, Monique A. M. Gignac, Gary Naglie, Jill I. Cameron

**Affiliations:** 1 Rehabilitation Sciences Institute, Faculty of Medicine, University of Toronto, 500 University Avenue, Suite 160, Toronto, ON Canada; 2 Dalla Lana School of Public Health, Institute for Work and Health, University of Toronto, 481 University Avenue, Suite 800, Toronto, ON Canada; 3 Department of Medicine and Institute of Health Policy, Management and Evaluation, University of Toronto, 3560 Bathurst Street, Room 278, Kimel Family Building, Toronto, ON Canada; 4 Department of Medicine, and Affiliated Scientist, Rotman Research Institute, Baycrest Health Sciences, 3560 Bathurst Street, Room 278, Kimel Family Building, Toronto, ON Canada; 5 Department of Occupational Science and Occupational Therapy, Rehabilitation Sciences Institute, Faculty of Medicine, University of Toronto, 500 University Avenue, Suite 160, Toronto, ON Canada

**Keywords:** Caregivers, Family centered care, Family caregiving, Patient-care, Patient education, Scoping review

## Abstract

**Background:**

Families play an important role meeting the care needs of individuals who require assistance due to illness and/or disability. Yet, without adequate support their own health and wellbeing can be compromised. The literature highlights the need for a move to family-centered care to improve the well-being of those with illness and/or disability and their family caregivers. The objective of this paper was to explore existing models of family-centered care to determine the key components of existing models and to identify gaps in the literature.

**Methods:**

A scoping review guided by Arksey & O’Malley (2005) examined family-centered care models for diverse illness and age populations. We searched MEDLINE, PsycINFO, CINAHL and EMBASE for research published between 1990 to August 1, 2018. Articles describing the development of a family-centered model in any patient population and/or healthcare field or on the development and evaluation of a family-centered service delivery intervention were included.

**Results:**

The search identified 14,393 papers of which 55 met our criteria and were included. Family-centered care models are most commonly available for pediatric patient populations (*n* = 40). Across all family-centered care models, the consistent goal is to develop and implement patient care plans within the context of families. Key components to facilitate family-centered care include: 1) collaboration between family members and health care providers, 2) consideration of family contexts, 3) policies and procedures, and 4) patient, family, and health care professional education. Some of these aspects are universal and some of these are illness specific.

**Conclusions:**

The review identified core aspects of family-centred care models (e.g., development of a care plan in the context of families) that can be applied to all populations and care contexts and some aspects that are illness specific (e.g., illness-specific education). This review identified areas in need of further research specifically related to the relationship between care plan decision making and privacy over medical records within models of family centred care. Few studies have evaluated the impact of the various models on patient, family, or health system outcomes. Findings can inform movement towards a universal model of family-centered care for all populations and care contexts.

**Electronic supplementary material:**

The online version of this article (10.1186/s12913-019-4394-5) contains supplementary material, which is available to authorized users.

## Background

Families play an integral role providing care to individuals with health conditions. As the number of individuals facing chronic illness continues to rise worldwide, there is a timely need to increase recognition of the care input made by family members. Nearly half of Canadians aged 15 years and older have provided care to persons with illness and/or disabilities [1]. Definitions of caregivers vary, but in general they are an unpaid family member, close friend, or neighbour who provides assistance with every day activities, including hands-on care, care coordination and financial management [2]. We use the term caregivers to reflect this definition. We use the term family to include the patient, caregiver(s) and other family members. When caring for patients with progressively deteriorating conditions and increasing care needs, over time, caregivers perform more complex care duties similar to those carried out by professional health or social service providers [3, 4]. Thus, caregivers play an important role in the care of persons with illnesses or disability across the entire illness trajectory.

Caregiving can be associated with negative outcomes. Caregivers’ physical and mental health, financial status, and social life are often negatively impacted, regardless of the care recipients’ illness [2–4]. As a result, the quality and sustainability of care provision at home may be threatened [5, 6]. Policies and programs to help sustain the caregiving role may reduce the negative consequences of caregiving and optimize care provision in the home.

The need to support caregivers to minimize negative outcomes and optimize the care they provide has received considerable attention in recent policy initiatives. In 2007, the Ontario Ministry of Health and Long-Term Care (MOHLTC) announced the Aging at Home Strategy, “to enable people to continue leading healthy and independent lives in their own homes” [7]. The strategy implies a shift away from institutional (e.g., long-term care) care towards home care, using population-based funding allocations to offer health and social services to seniors and their caregivers [7]. This is similar to initiatives in other provinces, such as British Columbia’s Choice in Supports for Independent Living (CSIL) program, introduced in 1994. As these strategies place increasing demands on caregivers, a broader initiative, the National Carer Strategy launched in 2008 and updated in 2014 articulates the need for universal priorities to support caregivers [8]. Changes in Canada are echoed in other countries, including Sweden and Vietnam [9–11]. These initiatives aim to facilitate a collaborative action plan to support seniors through policy.

Family-centered care has been proposed to address the needs of not only the patient, but also their family members. To date, family-centered care has been defined by a number of organizations. The Institute for Patient- and Family-Centered Care (IPFCC) [12] defines family-centered care as mutually beneficial partnerships between health care providers (HCPs), patients, and families in health care planning, delivery, and evaluation. Alternatively, Perrin and colleagues [13] define family-centered care as an organized system of healthcare, education and social services offered to families, that permits coordinated care across systems. In palliative care, family-centered care is defined by Gilmer [14] as a seamless continuity in addressing patient, family, and community needs related to terminal conditions through interdisciplinary collaboration. In the broadest scope, the notion of family-centred care embraces the view of the care-client as the patient and their family, rather than just the patient [14].

Building upon existing definitions, models of family-centered care have been proposed for a number of patient populations. The family-centered approach to healthcare delivery, developed most notably for pediatric-care, values a partnership with family members in addressing the medical and psychosocial health of patients. Parents are considered experts concerning their child’s abilities and needs [15, 16]. In the context of critical care, family-centered interventions may decrease the strain of caregiving in families during a crisis [17]. In the context of stroke, a family-centered approach to rehabilitation showed an improvement in adult children caregivers’ depression and health status one-year post stroke [18]. Other researchers have argued that family-centered care offers an opportunity to support families and strengthen a working partnership between the patient, family, and health professionals during end of life care [19]. With an aging population and a growing number of people living with chronic illness, family-centered care can help health care systems to provide support and improve quality-of-life, for patients and their families.

To date, there has been no synthesis of key components of original family-centered care models across all illness populations. Therefore, the objective of this paper was to conduct a scoping review of original models of family-centered care to determine the key model components and to identify aspects that are universal across illness populations, and care contexts and aspects that are illness- or care-context specific. This paper also aimed to identify gaps in the literature to provide recommendations for future research. A scoping review was selected as optimum because its goals are to generate a profile of the key concepts in the existing literature on a topic and identify gaps in the literature [20].

## Methods

We used a scoping review methodology guided by Arksey & O’Malley [21] to gather and summarize the existing literature on family-centred care models.

### Search strategy

A literature search of MEDLINE (including ePub ahead of print, in process & other non-indexed citations), CINAHL, PSYCHInfo and EMBASE databases was conducted. The search terms “family-centered”, “family-centred” were applied. The search strategy utilized a narrow focus due to the high degree of noise additional keywords generated. All searches were limited to English language publications from 1990 to August 1, 2018. We did not limit the patient population as we believed that the expected number of models in any sub-set of populations would be low. Searches were conducted by an Information Specialist. EndNote was used to organize the literature and assist with removal of duplicates. See Additional file 1 for an example of the search strategy.

### Inclusion and exclusion criteria

#### Inclusion criteria

- The focus of the article was on the development and/or evaluation of a family-centered model or a family-centered service delivery intervention
- The article considered healthcare providers’ communication/interactions with patients and families
- The focus of the article was on the development of a family-centered model in any patient population and/or location of care (i.e., acute care hospital, inpatient rehabilitation, community, institutional long-term care).

For the purposes of this paper, we used The Agency for Clinical Innovation (ACI)‘s definition of a model of care: “the way health services are delivered” ([22], p., 3). The definition includes where, by whom and how the intervention is delivered.

#### Exclusion criteria

- The focus of the article was an assessment or the evaluation of a tool that measured the degree of family-centeredness of a program, intervention or setting and not the evaluation of an original family-centered care model
- The article considered only interactions between family members
- The article was a review paper that did not propose an original model of family-centered care
- The article pertained primarily to ethical issues or the theoretical understandings of family-centered care
- The article was focused on training healthcare providers on how to deliver family-centered care and did not offer an original model of family-centered care.
- The article reviewed or discussed only the history, implications or rationale for family-centered care (e.g., the study conclusions suggested the need for a model of family-centered care)
- The article described a patient-centered care model that only included discussion of family interactions
- The focus of the article was on the development of a family-centered model exclusively for social support, rather than in a clinical, health-care context.

### Study selection and charting the data

The search identified 14,393 papers. One of the authors (K.M.K.) reviewed the citations and abstracts using the inclusion and exclusion criteria. Two other individuals reviewed 30% of the retrieved abstracts. Any discrepancies were discussed until consensus was reached. The inclusion/exclusion criteria were modified as needed to enhance clarity (final inclusion/exclusion criteria aforementioned). Full-text articles of all potentially relevant references were retrieved, and each was independently assessed for eligibility by one of the authors (K.M.K.) and the two other reviewers. There was 100% concordance between all reviewers on this second step. Reference lists of the included articles were reviewed by one of the authors (K.M.K.) and the two other reviewers, but no additional articles were added to the review.

Data were extracted by the lead author (K.M.K.) and the abstracted data content was reviewed for accuracy by the two other reviewers. Data were extracted relating to elements of the family-centered care model including details of the targeted population, objectives, intervention (if applicable), key findings and desired outcomes of the model. The key components of the models were systematically charted using a data charting form developed in Microsoft Word with a priori categories to guide the data extraction (see Additional file 2**).**

### Data synthesis

The purpose of this scoping review was to aggregate the family-centred model descriptions and present an overview of the key elements of the models. Methodological rigour of the publications was not examined, as consistent with scoping review methodologies [21].

The extracted data were collated to identify key components of models of family-centered care. K.M.K extracted the data and J.I.C reviewed the extracted data. K.M.K and J.I.C then followed qualitative thematic analysis using techniques of scrutinizing, charting and sorting the extracted data according to crucial nuances of the data, and this was summarized into descriptive themes characterizing model components [21, 23]. Data were synthesized using summary tables with tentative thematic headings. Themes and potential components of family-centered care models were discussed between K.M.K and J.I.C until consensus was achieved, and final themes were developed. When discussing themes, K.M.K and J.I.C analyzed the data within and then across different patient populations (diagnoses), age groups (pediatric vs. adult literature), and care contexts (e.g., acute care, community care) to identify aspects of models that are universal (i.e., do not differ across populations and care contexts) and aspects that are illness-specific. Final themes were then discussed with all authors. These themes represent answers to our study objective.

## Results

**Fig. 1:**
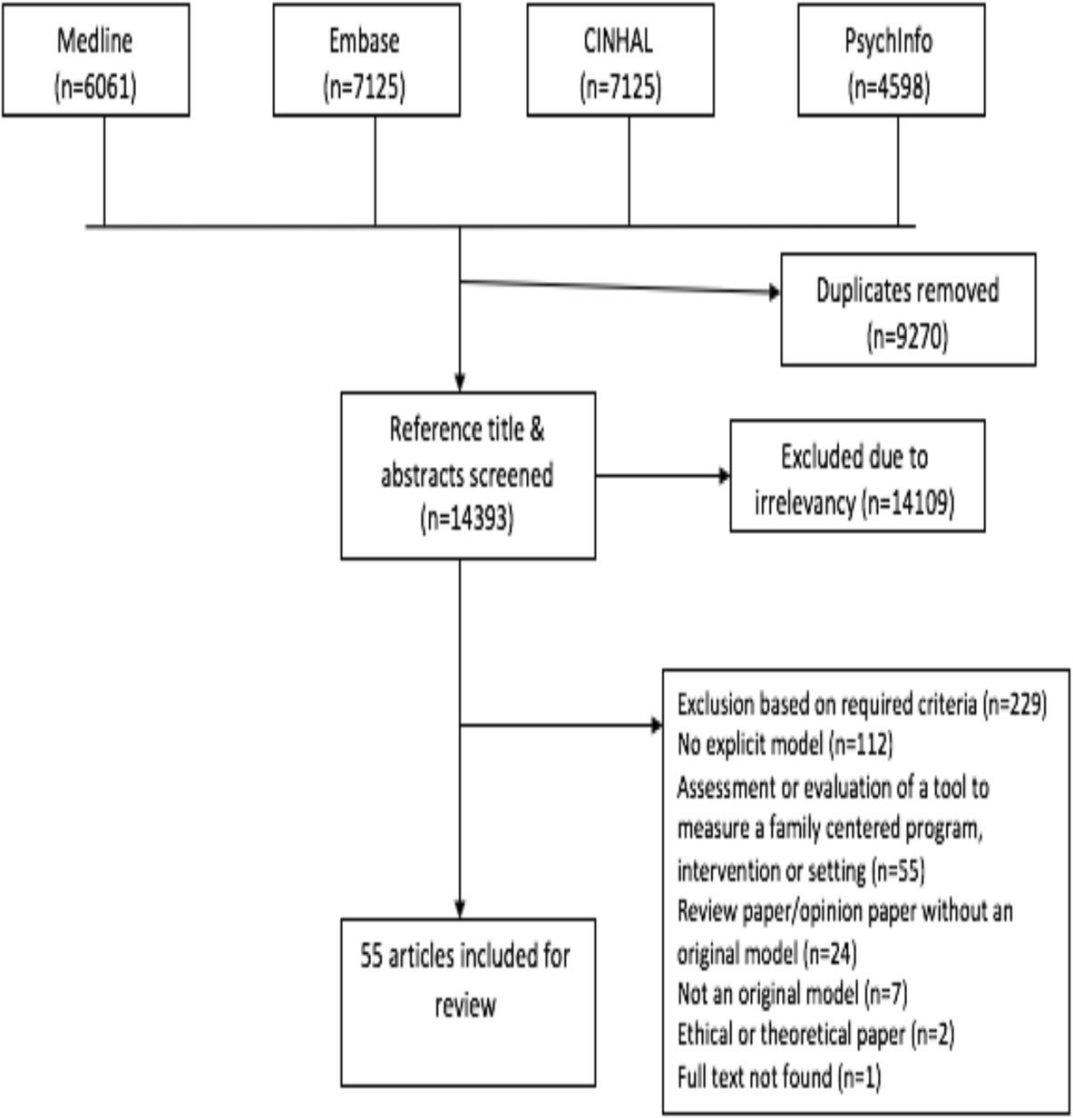
Search Selection

Fifty-five articles were included in this review (see Fig. 1). The majority of articles were not grounded in empirical research but proposed models for family-centered care and offered practical suggestions to inform implementation (91%). Of the 9 empirical studies, four articles (7%) described randomized control trials (RCTs) and one (2%) described a pre-test, post-test evaluation of their model. Three of the models tested in RCTs were developed to support families at the end of the patients’ lives, with the other model focused on behavioural change as part of childhood obesity treatment. The article describing a pre-test, post-test evaluation aimed to reduce alcohol and drug use through positive peer and family influences. Another study used longitudinal experimental quantitative design to measure the impact of their family-centered care model over two time points, using t-test analysis [24]. These studies demonstrated benefits of the models including enhanced feelings of mastery and empowerment for family members in care planning through skill building [24–26].

Two articles (3%) examined the feasibility of applying the concepts of family-centered models into practice. Eight articles (14%) described case studies of an implemented model that included families and children with complex medical needs in palliative and sub-acute settings with conditions including HIV, cancer and stem-cell transplantations. Another described patients and families in cardiac intensive care units (CICU) after operative procedures. Nine (16%) articles were literature reviews on FCC that lead to the description of a single hypothetical family-centered care model, but did not review all existing FCC models. One article (2%) compared HCPs’ roles in traditional models of care with their roles the family-centered model.

Out of the 55 included papers, 40 (73%) were explicitly designed for pediatric care recipients. In the pediatric literature, family-centered care models were proposed for a variety of populations which included, but were not limited to, cancer (*n* = 5), AIDS/HIV (*n* = 3), motor dysfunction (e.g. cerebral palsy) (*n* = 3), non-illness specific disabilities (*n* = 2), obesity (*n* = 3), asthma (*n* = 1), oral disease (1 paper), trauma (*n* = 2), autism (*n* = 1) and transplant recipients (*n* = 1). Eighteen studies did not report on a specific population. In adult populations, models have been presented for palliative care (*n* = 3), heart failure (*n* = 1), mental health (*n* = 1), cancer (*n* = 1), age-related chronic conditions (*n* = 1) and unspecified populations (*n* = 8).

Many of the family-centered care models have been developed for a variety of care contexts (e.g., community, acute care) and incorporate a variety of health care professionals. Care contexts included home/community care, acute hospital wards, emergency departments, critical care units, inpatient rehabilitation units and palliative care units. Professionals identified in the models primarily included nurses, social workers, physicians and nutritionists. In some models, psychologists, rehabilitation therapists and chaplains also were included. The core elements of FCC models did not differ by diagnosis, age or care context. This suggested that some aspects of FCC models were universal. We use the term “universal” to refer to this notion of high-level concepts that can be applied across illness populations, ages and care contexts. Universal and illness-specific aspects of FCC will be discussed in detail below.

**Fig. 2:**
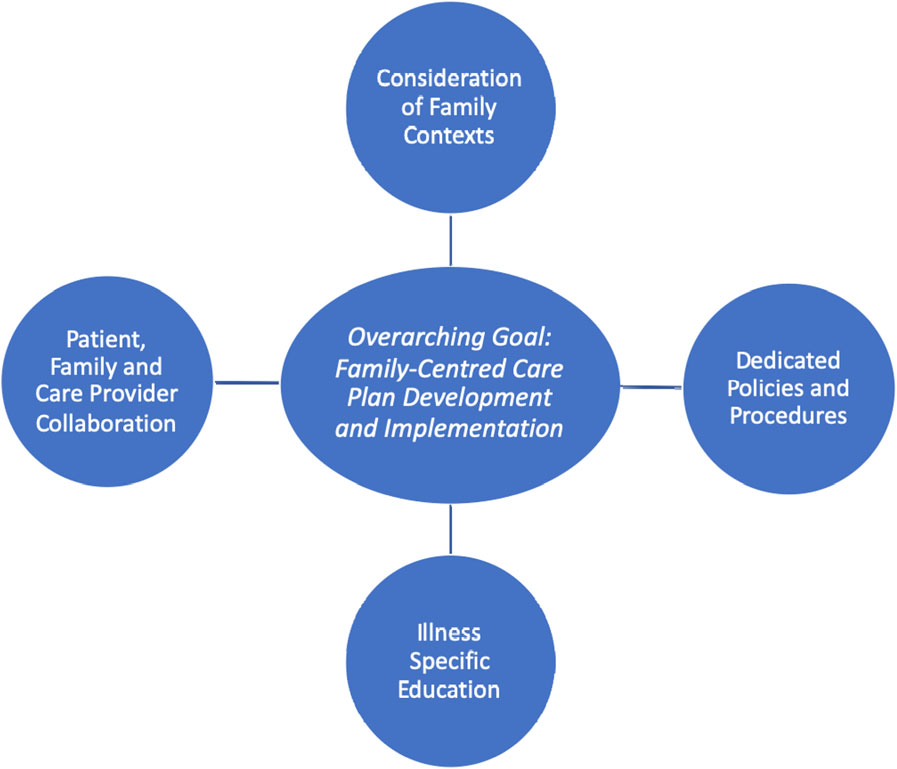
Universal Model of Family-Centered Care

Thematic analysis revealed a universal goal of FCC models to develop and implement patient care plans within the context of families. To facilitate this aim, family-centered care models require: 1) collaboration between family members and health care providers, 2) consideration of family contexts, 3) education for patients, families, and HCPs, and 4) dedicated policies and procedures. Figure 2 provides a graphical overview of the key components of FCC. This figure highlights the overarching goal of FCC models and the key components required to help facilitate this goal including both universal and illness-specific components.

### Overarching theme: family-centred care plan development and implementation

This overarching theme describes the universal goal of family-centered care models to develop and implement patient care plans that are created within the context of unique family situations.

All 55 models supported the development of family-centered care plans with specific short and long-term outcomes [27–29]. Patients, families and HCPs were considered key partners who should contribute to the clarification of care plan goals [30–33]. Care plans should consider the day-to-day ways of living for patients and families [34–40] by encouraging the maintenance of home routines [41]. Potential goals identified included achieving family and patient identified functional milestones like a new motor skill (e.g., running) [30]; decreasing delays or complications at hospital discharge [42]; improving patient and family satisfaction with care [40–42]; and improving caregiver support [43]. Patient involvement in care plans was acknowledged as conditional upon the patient’s capacity to participate (it may exclude young children, people with illnesses affecting their cognition, etc.). From the initial diagnosis, the models championed that all members of the patients care team should elicit families’ perspectives regarding priorities, families’ needs and concerns, and their abilities to provide care [14, 29, 37, 44–48]. Understanding families’ needs and priorities was deemed important and as contributing to realistic and better-defined outcomes [24, 45], as well as important to enhance families’ abilities to support the plan and optimize patient outcomes [41, 49–51].

The models also emphasized that HCPs and family members should share in the implementation of the care plan. Care delivery should commence when everyone is in agreement with the care plan [42]. It was noted that family members should be encouraged to communicate any issues or priorities they have regarding care to HCPs [27, 44]. Family members of relatives who are inpatients also should be involved in discharge planning, such as describing their concerns and ability to perform care duties [42] so as to troubleshoot and optimize care in the community. Some models noted that care plans can be made achievable by breaking them down into smaller steps for the family [27]. One model also hypothesized that monitoring the success of the care plan has the potential to optimize service use by bridging service gaps and eliminating any duplication of resources [52].

### Model components

In addition to the overarching theme related to care plan development and implementation (described above), other key model components needed to facilitate family-centered care plans were identified. These encompassed collaboration and communication; education and support; consideration of the family context; and the need for policies and procedures.

### Collaboration

Family-centered care models highlighted collaboration between HCPs, patients and families as key in the development of care plans. Collaboration was noted as being required across the illness and care trajectory [53] to enhance patients’ and families’ abilities to maintain control over the patient’s care plan and delivery [54], particularly as care becomes increasingly complex [55].

Many of the family-centered care models offered some insight into how families and HCPs could work together in the delivery of care across the care trajectory. Trusting [54], caring and collaborative relationships [44] between families and HCPs were identified as key and efforts should be undertaken to cultivate them. In this collaborative relationship, HCPs were encouraged to relinquish their role as a single authority. Some authors even argued that families should have decision-making authority to decide upon the role and the degree of involvement of HCPs in developing care plans [28]. Attharos and colleagues [55] further suggested that family-centered care models should have defined roles for each family member, the patient, and all involved HCPs. All roles are essential to the development of care plans [56].

#### Communication

Family-centered care models were thought to better facilitate communication and exchange of information and insights among family members, patients and HCPs related to the development and delivery of care plans [54]. One model suggested that clinicians should offer care options and patients and families should advocate for patient preferences and family values [57]. The exchange of information was encouraged to be open, timely, complete and objective [27, 55].

The models encouraged HCPs to use a variety of strategies to communicate with and support caregivers and patients, including interdisciplinary care and diagnostic reports, community follow-up in-person or by virtual meetings, and resource notebooks listing community supports to help care for the patient [29]. HCPs were also encouraged to communicate disease-specific information to help patients and family members make appropriately-informed disease-related decisions [32, 39, 57].

### Education

Education about care provision and the disease was deemed necessary to enable family-centered care. Education was typically approached from the concept of mutual learning, whereby patients, family members and HCPs all learn and support each other [38, 54]. In general, models advocated for training of HCPs to effectively elicit information and communicate with patients and family members. This was thought to decrease anxiety and increase control for patients and caregivers [14, 32, 58, 59].

Various researchers posited ways to best provide education about the illness and care provision to patients and families. Connor [60] suggested that written information, describing the family-centered approach to care should be provided to patients and caregivers. Further, all information should be presented at a language level that is understandable to the family and patient [42, 43, 60], which means minimizing technical (e.g., medical) jargon [59].

Education was though to be ongoing, meaning that it often doesn’t end when a patient is discharged from an inpatient setting. Models highlighted a need to establish methods to continue educating caregivers beyond inpatient care [49], such as by providing the patient and family with a follow–up care plan after discharge and possibly follow-up contact by HCPs [33]. Appropriately communicated education is believed to foster a sense of trust [42] and help patients and family members become more knowledgeable. As a result, patients and families were thought to become more independent in making informed treatment decisions [14, 35, 37].

Some models noted that patients and their family members find it helpful to learn from other patients and caregiver peers [32]. Peer sharing of mutually supportive resources and other experiences related to living with an illness or providing care can serve as an important means of improving knowledge about support resources [32] and enhancing emotional support [60, 61]. This was thought to be possible as part of family centered care models by care teams helping to cultivate friendships and general peer support with other families in similar caregiving situations (for example, those caring for care recipients with the same illness). Other mechanisms to offer peer support could include patient and caregiver support groups, workshops, group retreat trips, and shared respite care [14].

#### Family support needs

Family members may experience a negative impact on their own well-being as part of the ongoing demands of caregiving. Recognizing that families are often psychologically stressed and can have difficulties coping [59], family-centered care models emphasized support for family members’ well-being [62]. Supporting families often included emotional support and providing education and training on care delivery that takes into account caregiver needs and preferences. Family-centered models acknowledge that caregivers are experts in matters concerned with their own well-being [47]. HCPs were thought to support caregivers’ by providing education to foster caregivers’ confidence in their ability to provide care and develop care plans.

Family-centered care models emphasized that care recipients function best in a supportive family environment [41]. Identifying the impact of the illness on the patient and the family is crucial to providing emotional support [42]. At a minimum, information about support needs should be gathered from both patient and family [63]. Particularly in pediatric patient populations, authors spoke of identifying types of support that are based on the patient’s developmental capacities [64] and needs, while also taking into consideration the social context of the patient and family’s life [48]. Support needs can be determined through well-designed, semi-structured activities, including questionnaires [48] and discussions with the family.

Health systems utilizing a family-centered care model were thought to help sustain caregivers by providing them with resources to support their caregiving activities [65]. Goetz and Caron [49] state that organizational support should make existing health service community resources more family-centered by considering the family in all aspects of program delivery. Topics that need to be addressed by services offered to family members included, mental health, home care, insurance/financing, transportation, public health, housing, vocational services, education and social services [13].

### Consideration of family context

Family was conceptualized in different ways across models. For example, some models described including the family-as-a-whole (every family member, not just those that provide care) [63, 64], whereas others described the family as those who provide care [44]. Authors highlighted that families have ‘the ultimate responsibility’ [63] and should have a constant presence throughout the care and illness trajectory [60]. Consistently across family-centered care models, families were seen as vital members of the care team [38] who provide emotional, physical, and instrumental levels of support to the patient [32, 54].

#### Family strengths

Three of the models underscored that family-centered care is based on a belief that all families have unique strengths that should be identified, enhanced, and utilized [41, 53, 66]. Models identified various examples of family strengths in care delivery including resilience [41], coping strategies [58], competence and skill in providing care [25, 26] and motivation [49]. None of the models discussed how these strengths would be identified when implementing family centered care. Three of the models stated that family-centered care should continuously encourage caregivers to utilize these strengths [53, 57, 64] although specific examples of how to do so were not included. Identifying areas of weakness that may require education and training was not discussed in the models.

#### Cultural values

Families were thought to contribute to a culturally sensitive care plan by discussing their specific cultural needs, as well as their strengths related to personal values, preferences and ideas. Caregivers’ social, religious, and/or cultural backgrounds can influence the provision of care to their family member [29, 49, 57, 58]. One model suggested that HCPs need to elicit information about families’ beliefs [26] to help guide culturally sensitive care plans (e.g. religious participation). The process by which this would occur was not discussed in detail.

### Dedicated policies and procedures to support implementation

To support implementation, family-centered care models should have dedicated policies and procedures that are also transparent [56, 67, 68]. Both the macro and micro levels of society need to be considered when trying to implement family-centered practices [13]. Perrin and colleagues [13] described macro level issues as including government policies and agencies (e.g., national, provincial, municipal), while micro level factors include community service systems (e.g., physicians, other HCPs, schools, public transportation, etc.). Examples of macro-level considerations include incorporating families in nation-wide policy making and program development [29, 53, 56, 60]. Micro-level considerations include incorporating family members and patients in decision-making for local community organizations [69], the implementation of health programs and care policies at regional hospitals, as well as in HCP education [29].

Family-centered care policies were identified as important as they legitimize and support families’ contributions to the care of their family member. For example, in pediatrics, Regan and colleagues [39] suggested changing policy to open visitation hours to increase family members’ roles as partners in care. This could increase the number of interactions between HCPs, patients and families, and, as a result, further support caregivers in their caring role [24].

Family-centered care models also noted the importance of considering the physical environment when developing policies and practices. The physical environment of care settings should be created and tailored to meet the needs of patients and families [54], although concreate examples were not provided. Both the patient and family should be included in the development and evaluation of facility design [29], where possible, as well as modifications to the home environment.

## Discussion

The purpose of this scoping review was to identify core components of family-centered care models and to identify components that are universal and can be applied across care populations. This paper also aimed to identify gaps in the literature to provide recommendations for future research. Most models were developed for pediatric populations with a number of models emerging for the care of adult populations. The synthesis suggests that there are core components of family-centered care models that were not unique to specific illness populations or care contexts making them applicable across diverse health conditions and experiences. From a theoretical perspective, our review adds to our understanding of how FCC is conceptualized within the current state of the literature and suggests the possibility of moving towards a universal model of FCC. This includes developing a care plan with defined outcomes and that incorporates patient and family perspectives and their unique characteristics. This also includes collaboration between HCPs and family members and flexible policies and procedures. However, there were some aspects of models that were specific to illness populations such as illness-specific patient and family education.

Currently, person-centered care is considered best practice for improving care and outcomes for many illness populations. Patient-centered care has been described as being “respectful of and responsive to individual patient preferences, needs and values, and ensuring that patient values guide all clinical decisions” [70]. Person-centered care involves: acknowledging the individuality of persons in all aspects of care, and personalizing care and surroundings; offering shared decision making; interpreting behavior from the person’s viewpoint; and prioritizing relationships to the same extent as care tasks [71]. Aspects of patient and person-centered care were identified as key components of family-centered care models including focusing on patient and family values, preferences and needs, related to their own circumstances and family contexts. In addition, this review identified specific components that go beyond patient-centered care that are required to address the needs of families including focusing on respectful communication to facilitate the necessary patient/family-professional partnerships and collaboration needed to develop and implement care plans. Moreover, there is the need for the patient/family-professional partnerships to respect the strengths, cultures and expertise that all members of this partnership bring to the development and delivery of care plans.

Implementation of illness-specific models of care for multiple different illnesses may be challenging for health care systems. As many individuals live with multi-morbidity, a non-illness specific family-centered care model may meet the needs of more individuals [72]. Yet, there is a lack of discussion in the literature of concrete strategies to help implement the key concepts identified in our review. Moreover, the research on implementing FCC models in real world situations is scant. In order to encourage changes in health care systems there is a need for evidence that the concepts of FCC lead to improvements.

This review identified aspects of family-centered care that are illness-specific. Illness-specific education and support is required at each stage of the illness recognizing differences in illness trajectories across patient populations [73]. Currently, the models are described with static concepts that are not reflective of ongoing and changing illness trajectories. Providing illness-specific care, advice, and information that is sensitive to their place in the illness trajectory may greatly influence caregivers’ capacity to support the care recipient. Further research is needed to understand how family-centered care may evolve across the illness and care trajectory.

More research is needed to enhance the potential of a universal family-centered care model that crosses age groups, conditions, and care settings. For instance, in the current models of FCC, there is not consistent definition of what constitutes the family. In the pediatric literature the family primarily includes the parents of the child, but does not usually include siblings or extended family members who may be providing care. Moreover, current FCC models fail to address conflict and mediation for circumstances where there is the potential for family members to disagree with one another, with the patient or with the HCPs regarding care plans or other aspects of care.

As many of the models were developed for pediatric populations, they fail to acknowledge aspects such as privacy issues and cognitive capacity. These issues become relevant as we consider models of care for adult populations. In instances where patients’ cognitive capacity influences their ability to participate in decision-making, family caregivers become active contributors to care plan development and implementation. Caregivers can benefit from having access to patients’ medical information to contribute to treatment decision making and inform care and service use. Current privacy legislation does not automatically give families access to relevant information. Future research should explore adult patients’ preferences for family members to have access to their medical records, as their preferences will influence the management of privacy in models of FCC.

Lastly, the articles included in this review were primarily descriptive and not evaluative. Evaluations are needed to demonstrate the benefit of FCC to patient, caregiver and health system outcomes. Potential evaluation outcomes can include satisfaction with care, improvements in patient health and caregiver health and stress, and efficient use of health services. There should be consensus regarding outcome measures to be used when evaluating FCC models to enhance our ability to compare across models. Model evaluation is needed to provide empirical evidence to support or reject the concepts of FCC in both universal and illness-specific contexts. Moreover, we need methods to assess implementation of FCC in practice. For example, measures to assess the family-centered nature of care are being developed [24–26]. The review suggests we may need new assessment tools to assess, for example, family strengths.

Concrete steps are needed to implement a universal FCC care model into practice. While we have defined the components of a model, we have also highlighted additional empirical research that is needed to further define model components in real-world settings, within and across various care contexts and illness populations. In particular, we recommend the testing of our universal model in the context of a randomized control trial (RCT).

Lastly, we recommend the development of outcomes measures to determine if FCC leads to improvements in patient and family satisfaction, mental and physical health outcomes, enhanced efficiency, health system utilization (e.g., decreased length of hospital stay or return hospital visits), community reintegration and cost-effectiveness. Important outcomes should relate to families, patients of all ages and health care professionals. Outcome measures related to health care professionals may include enhanced comfort working with families and patients. Valid and reliable measures are essential for the evaluation of a model’s effectiveness and to translate FCC models into practice.

### Study limitations

This scoping review is not without limitations. Only published, English language articles were included, thus excluding other models that may exist in other languages. This may have also limited models to those that were developed and/or tested in predominantly English-speaking counties. We also did not explore grey literature, limiting our models to only those that underwent peer review. Many of the included models were designed for the pediatric population, so findings have limited application to adult populations.

## Conclusion

This paper used an established scoping review methodology to synthesize 55 models of family-centered care. We were able to determine the universal components of the models that place both the patient and family at the center of care, regardless of the patient’s illness or care context. Findings outline aspects of FCC that are universal and aspects that are illness specific. Universal aspects include collaboration between family members and health care providers to define care plans that take into consideration the family contexts. It also includes the need for flexible policies and procedures and the need for patient, family, and health care professional education. Non-universal aspects include illness-specific patient and family education. Future research should evaluate the ability of FCC to improve important patient, family, caregiver, and health system outcomes. Health care policies and procedures are needed that incorporate FCC to create system level change. Our review moves the field of FCC forward by identifying the universal and illness-specific model components that can inform model development, testing, and implementation. Advancing FCC has the potential to optimize outcomes for patients, families, and caregivers.

## Additional files

Additional file 1: Search Strategy Example (Medline). (DOCX 13 kb)

Additional file 2: Data Abstraction. (DOCX 90 kb)

## Data Availability

Not applicable.

## Abbreviations

FCC: Family-centered care
HCPs: Health care professionals
