# Supplementary material for: Towards a universal model of family centered care: a scoping review
Source: BMC Health Serv Res. 2019 Aug 13;19:564. doi: 10.1186/s12913-019-4394-5 (PMC6693264; doi:10.1186/s12913-019-4394-5)
Supplement: Supplementary file 1 — Search Strategy Example (Medline). (DOCX 13 kb) [file 12913_2019_4394_MOESM1_ESM.docx]

Additional file 1:

**Search Strategy Example (Medline)**

Database: Ovid MEDLINE(R) Epub Ahead of Print, In-Process & Other Non-Indexed Citations, Ovid MEDLINE(R) Daily and Ovid MEDLINE(R) <1946 to Present>

Search Strategy:

--------------------------------------------------------------------------------

1 (famil* adj focus*).ti,ab,kw,kf. (1132)

2 (famil* adj3 (cent?er* or cent?re*)).ti,ab,kw,kf. (6534)

3 1 or 2 (7584)

4 limit 3 to english language (7060)

5 4 not (exp animals/ not exp humans/) (7027)

6 limit 5 to yr="1990 -Current" (6061)
***************************
