# Supplementary material for: Towards a universal model of family centered care: a scoping review
Source: BMC Health Serv Res. 2019 Aug 13;19:564. doi: 10.1186/s12913-019-4394-5 (PMC6693264; doi:10.1186/s12913-019-4394-5)
Supplement: Supplementary file 2 — Data Abstraction. (DOCX 90 kb) [file 12913_2019_4394_MOESM2_ESM.docx]

**Family Centred Care – Data Extraction**

| **CITATION** | **Article Type/ Study Design** | **Model Name** | **TARGET POPULATION/LOCATION/ INVOLVEMENT** | **COMPONENTS** |
| --- | --- | --- | --- | --- |
| Ahmann, E., & Bond, N. J. (1992). Promoting normal development in school-age children and adolescents who are technology dependent: a family centered model. *Pediatric Nursing, 18*(4), 399-405. | Model Development | *Possibly:* Family Centred Approach to Developmental Assessment and Intervention | **Pediatric -**School-age children and adolescents who are dependent on or assisted by medical technology  **Where:** In a medical facility or in the community  **HCP Involved**: Nurses | **Objectives:**   - Normalize the life experiences of school age children and adolescents who are dependent on or assisted by technology   **Intervention/Process**   - Interview the family and the child (privately if an adolescent) - Sharing complete and unbiased information with families - Recognizing the strengths of family members and family as a whole - Helping families to build on those strengths keeping in mind diversity in goals and priorities that families have for their children - Emphasis on collaboration and working through differing priorities of family and nurse even if the family or adolescent chooses goals that are medically or socially risky   **Desired Outcome:** Make the accomplishment of developmental tasks easier.  **Other notes:** Families have the ultimate responsibility in promoting a child’s development, and family-centred nurses can support them in several ways through this process. |
| Ainsworth, F. (1998). Family centered group care practice: Model building. *Child & Youth Care Forum, 27*(1), 59-69. doi:http://dx.doi.org/10.1007/BF02589528 | Evaluation of a model | *Possibly:* Family-Centered Approach to Group Care | **Pediatric-** children in group care and their families.  **Where:** Group care settings  **HCP Involved:** Social workers (Group Care Practitioners?) to be a parent educator, trainer, supporter | **Objectives:**  To improve group care at policy, organization, and practice levels so that the best interests of the child and family are recognized and effectively dealt with.  To move away from blaming parents to collaborating with them.  **Intervention/Process:**   - Organizational change: getting child and youth workers on board with a family-centered perspective (not blaming parents, understanding parents are also psychologically stressed and unable to cope) - Identify learning styles of parents and children (observational, shared participation, or combination of both) - Use real-life situations and events to engage parents and to teach child-rearing and nurturing skills   **Desired Outcome/ End Goal:** To preserve, and wherever possible, to strengthen connections between children in placement and their birth parents and family members  **Other Notes:** Group care has historically been regarded as ineffective and incapable of reforming. There is a need to incorporate birth parents and family members as partners in the care and treatment process.  Change needs to occur at the organization, policy and practice level. Also, birth parents and family members do not have adequate coping skills and strategies to deal with environment stress and problem behaviours – these can be taught and/or modeled by group care staff.  Author offers seven areas of child and youth worker activities and skills (Not for families)   - - Organization of the care environment   - Team functioning (positive involvement, shared responsibility)   - Activity programming   - Working with groups   - On the spot counseling (immediate counseling support when participants have challenging behaviours)   - Use of everyday life events (teach participants real life skills)   - Developmental scheduling (assess needs of participants based on developmental stage, teach new skills that allow them to advance confidently) |
| Attharos, T., Khampalikit, S., Phuphaibul, R., & Tilokskulchai, F. (2004). Development of a family-centered care model for children with cancer in a pediatric cancer unit. | Model Development; Case Study | *Possibly:* A model of family-centered care for the children with cancer in a pediatric cancer unit | **Pediatric-** Children with cancer  **Where:** Pediatric cancer units in a large hospital (which delivered medical care to patients with low immunity from 1 to 15 years of age)  **HCP Involved:** Nurses | **Objectives:**   - To provide nursing care based on the concept of family-centered care as a standard, modify practice and increase self-esteem and professionalism   **Desired Outcome/End Goal:** To allow families to openly and continuously communicate with nurses, being confident in caring for their children and satisfied with care  **Intervention/Process:**  Four guiding principles of the family-centered care model:   1. caring and empathetic relationship: interaction between nurses and child/family (spending time together, caring, being empathetic and showing respect) 2. mutual learning: exchange of information, advice and feedback between nurses and families 3. partnership: participation of the family in caring for the child, mutual decision and coordination between nurses and family 4. enhancement of strength: includes spiritual needs, family’s abilities, and development of the support system   Process of care :  1. Stage one - Initiation of family-centered care: development of a trusting relationship between nurses and families and mutual learning the first few days post-admission   - *Nurse’s roles:* establishing rapport with the family, enhancing spiritual strengths, learning about child and family, providing information and encouraging family participation in basic care - *Family’s roles:* open communication with nurse, comprehension of information/instructions and participation in basic care - *Child’s roles:* adhering to treatment plan, performing self care as able   2. Stage two - Mutual care: enhancement of collaboration *(families able to provide more complicated care for the child)*  3. Stage three - Independent care by the family: child has been admitted several times, family strengths enhanced by providing instrumental and emotional support, assistance from nurses only for complicated treatment  **Other Notes:** The rational is that by collaborating with nurses through family-centered care, families develop additional skills required to meet the needs for the well being of a child with cancer. By being involved in care, families also increase their own coping abilities and confidence. |
| Baker, J. N., Barfield, R., Hinds, P. S., & Kane, J. R. (2007). A process to facilitate decision making in pediatric stem cell transplantation: the individualized care planning and coordination model. Biology of Blood & Marrow Transplantation, 13(3), 245-254. doi:https://dx.doi.org/10.1016/j.bbmt.2006.11.013 | Model Development ; Case Study | Individualized Care Planning and Coordination (ICPC) Model | **Pediatric-** children undergoing stem cell transplantation (SCT)  **Where:** Any facility that cares for children undergoing stem cell transplantation.  **HCP Involved**- those who are involved with children’s’ stem cell transplantations. | **Objectives:**   - To facilitate and enhance the ethical and effective decision making for both the health care team and family members.   **Desired Outcome/End Goal:** ICPC provides a framework to allow for attending to cure-oriented focus of transplantation in addition to attending to the psychosocial experiences of the child and family.  **Intervention/Process::** A 3-step model based on a foundation of competence, empathy, compassion, communication and quality   1. Relationship – understanding the illness experience from the perspective of the patient and family, sharing relevant information, and assessing ongoing needs 2. Negotiation – prognosticating, establishing goals of care, and discussing treatment options 3. Plan – generating a comprehensive plan of care that includes life and medical plans   **Other Notes:** Care decisions are likely to be both disease-directed and informed by family and patient preferences. The likelihood of significant suffering, the high incidence of morbidity and mortality, and the need to make frequent difficult decisions in an ethical and effective manner make ICPC an effective model for this population. |
| Biggert, R. A., Watkins, J. L., & Cook, S. E. (1992). Home infusion service delivery system model: a conceptual framework for family-centered care in pediatric home care delivery. *Journal of Intravenous Nursing, 15*(4), 210-218. | Model Development | Home Infusion Service Delivery System Model. | **Pediatric:** This model targets children who require home health care.  **Where:** Home health care and related service delivery systems. Written from Washington state context. Generalisability might be limited to the USA due to elements of the model that are limited to the American health care system (i.e. Reimbursement specialist)  **HCP Involved:** Physician, home care nurse, and IDS (I.V. nurse, reimbursement specialist, pharmacist). | **Objectives:** To clarify the health care provider’s goals and professional relationships for themselves and the family. To assist families in meeting their self-identified health goals for the child.  **Process/Intervention:** Comprised of four interdependent service delivery systems, a physician, a family system and a communication system. Each of the roles is clarified and the model provides a depiction of the ways these systems interact. TQM is the communication system that ensures positive outcomes by putting in place mechanisms for evaluation and improvement so that seamless service delivery is possible. |
| Beers, L. S., & Cheng, T. L. (2006). When a teen has a tot: a model of care for the adolescent parent and her child. Contemporary Pediatrics, 23(4), 47-56. | Model development; Literature Review | The Teen-Tot Model of Family-Centered Care | **Adolescent Parents-** This model targets adolescent parents and their child(ren)  **Where:** Can be implemented elsewhere. Already implemented. Teen-tot program has been implemented at The Healthy Generations Program in Washington, D.C.  Program in Washington, D.C. is multifaceted – primary care, case management services (referral coordination, assistance with return to school, career counseling, crisis management), developmental assessment, social work (housing assistance, crisis intervention), psychologist (mental health screening and counseling service), and home visits for parenting support.  **HCP involvement**: none specified. Those involved with teenage parents. | **Objectives:** To incorporate family-centered principles through the continuum of care – support through pregnancy, childbirth, infancy, childhood, adolescence, and young adulthood  **Desired Outcome/End Goal:** To effectively navigate the challenges associated with being a teen parent, including health risks for the child, and educational and financial hurdles of parents  **Intervention/Process:** Using the below FCC principles, with focus on organization, education, and behaviour modification:   - Respect each child and his/her family - Honour racial, ethnic, cultural and socioeconomic diversity - Recognize and build on the strengths of each child and family - Facilitate choice about approaches to care - Be flexible in policies, procedures, and provider practices (tailor to needs of patient and family) - Share useful information - Ensure support from pregnancy to caring for child as a young adult - Collaborate with families in the care of the individual child, in policy making, and in program development - Empower each child and family to discover their own strengths, build confidence and make choices about their health (Adapted from American Academy of Pediatrics Committee on Hospital Care and Institute of family Centered Care   **Other Notes:** FCC helps young parent see how her physical and emotional health and that of her baby are interdependent.  Effective care is:   - Provide a medical home for teenage parents and their children - Address the development of both infant and teenage parent - Encourage continuation of healthful behaviours - Assess for risk of domestic violence - Emphasize to the parent the importance of completing high school - Include both parents when possible - Stress the importance of adolescent caring for the child even if others are involved - Utilize community resources - Provide positive reinforcements to successes (ie. Avoiding substance abuse, continuation of breast feeding, achieving educational goals, etc) |
| Brady, M. T., Crim, L., Caldwell, L., & Koranyi, K. (1996). Family-centered care: a paradigm for care of the HIV-affected family. Pediatric AIDS & HIV Infection, 7(3), 168-175. | Model Development | *Possibly:* The Family AIDS Clinic and Educational Services | **Pediatric/Parents-** Children and parents with HIV/AIDS  **Where:** A pediatric hospital with a large area that provides care to HIV positive children  **HCP involved:** Those that are involved in the treatment of HIV Positive children: Physicians, nurses, social workers, nutrition specialist, psychologist, child-development specialist, dentist, and chaplain | **Objectives:** To address the needs of all family members as a unit by providing comprehensive, coordinated, culturally sensitive, family-centered care to HIV-infected children and their affected family members at one location  **Desired Outcome/End Goal:** The goal is to reduce service duplication and accessibility barriers.  **Intervention/Process:**  Medical care – Patient (child & parents) activities are coordinated, care is received simultaneously at clinics shared by both pediatric and adult health care providers. Time required for services is reduced by doing the laboratory work in the clinic instead of the outpatient lab. Measures to increase compliance include child care, evening clinic hours, transportation assistance, support groups, and home care services.  Psychosocial services – Initiation of a parent support group, women’s support group, and a siblings treatment curriculum to aide families with the disclosure of an HIV/AIDS diagnosis to other family members and friends. These groups also make efficient the use of services, increase community interaction, and promote collaboration.  Case management – Bridge service gaps by coordinating care through an interdisciplinary team. At weekly meetings, individual and family care plans are reviewed and case managers coordinate appropriate treatment, services, and informational support. Home visits are made to assess resources and community supports.   1. Identification of eligible families 2. Performance of a needs assessment 3. Development of a care plan 4. Identification of available service providers 5. Identification of services not currently available at our program or in the community 6. Service referral and coordination 7. Follow-up and reassessment of adequacy at meeting client needs   **Other Notes:** HIV is an unpredictable course and leaves the patient with complex/diverse service needs that change over time. HIV is increasingly affecting children, adolescents, women, and families, thus, the rationale behind FCC is that health care needs to be organized and delivered in ways that support the entire family. |
| Brown, K., Mace, S. E., Dietrich, A. M., Knazik, S., & Schamban, N. E. (2008). Patient and family-centred care for pediatric patients in the emergency department. *CJEM Canadian Journal of Emergency Medical Care, 10*(1), 38-43. | Model Development; Literature Review | Patient and Family-Centred Care (PFCC) | **Pediatric**  **Where:** Can be applied in any size hospital emergency department.– guidelines for implementation are provided.  **HCP Involved:** Family and health care practitioners collaborate to determine child’s role in medical decision-making (based on stage of development and capacity to participate) | **Objectives:** To implement principles of PFCC in order to improve quality of care.  **Desired Outcome/End Goal:** Believed that PFCC results in increased satisfaction with care.  **Intervention/Process:**  Principles of PFCC:   1. Treat patients with dignity and respect 2. Communicate unbiased information 3. Patient and family participate in experiences that enhance control and independence and build on family strengths 4. Collaborate in the delivery of care, policy and program development and professional education   PFCC with pediatric patients in the emergency department   - Family member presence in patient care area, even during procedures and resuscitation (if desired) - Shared decision making among pediatric patient (when appropriate), family and health professionals   Steps in implementation:   1. Determine need 2. Evaluate policies and procedures for PFCC principles 3. Establish a unified departmental and hospital philosophy 4. Educating staff 5. Instituting and modifying policies and procedures 6. Assessing the current environment and making modifications (including identifying potential barriers to implementation) |
| Byers, J. F. (1997). Holistic acute care units: partnerships to meet the needs of the chronically ill and their families. AACN Clinical Issues, 8(2), 271-279. | Model Development; Literature Review | H Holistic Care Model | **Chronic Condition Patients-** Chronically ill patients suffering from different complex medical conditions such as AIDS, heart failure, and COPD, as well as their families.  **Where:** This model has been applied at Orlando Regional Medical Center. A unit in acute care hospital that is specifically designated as a “Holistic Care Unit.”  **HCP Involved:** Unit-based multi-disciplinary team: Nursing, social worker, dietician, recreational therapist, and chaplain. | **Objectives:** To address physical, mental, emotional, and spiritual health to optimize healthcare experiences of both patient and family.  **Outcome/End Goal:** To decrease anxiety and enhance coping (using complementary therapies). Also, long-term management ought to be the goal, recognizing patient as an integral part of the family unit.  **Process/Intervention:** A synthesis of the best elements of family-centered care, cooperative care, and the Planetree model.  Elements of Holistic Care:   1. Family viewed in its social, cultural and religious context 2. Person is viewed holistically 3. Active involvement of patient and family in care planning (shared decision making) 4. Provision of explicit information to enable decision making and educate family and patient re: the disease and its management 5. Negotiation of patient and family involvement in technical care routine 6. Complementary therapies should be available – and accessed independently 7. Encouragement of maintaining home routine as much as possible 8. Consideration of impact of disease on patient and family and support given as appropriate  - Services include assessment, counseling, facilitation, and education - Emphasizes empowerment, humanizing the experience, family involvement, self-management, communication, connectedness and “being there” for the patient   **Other Notes:** Traditional acute care health care delivery has a goal of resolving a medical crisis and therefore, does not meet the needs of chronically ill patients and their families because this population is not amenable to a “cure” and thus does not fit the traditional medical model.  Synthesis of the best components of patient focused care models is needed. |
| Callahan, H. E. (2003). Families dealing with advanced heart failure: a challenge and an opportunity. Critical Care Nursing Quarterly, 26(3), 230-243. | Model Development | *Possibly:* A Model for Working with Families Dealing with Advanced Heart Failure *(based on Friedman Family Assessment Model within the context of the Resiliency Model of Family Stress, Adjustment, and Adaptation).* | **Patients with Heart Failure-**and their families.  **Where:** Any care setting, so long as care plan can be transferred to other settings.  **HCP Involved:** Nurses | **Objectives:** To assess key areas of family life in a structured manner  **Desired Outcome/End Goal:** Could resolve the issue of discomfort felt by nurses in assessing and working with the family unit through an established structure for dealing with families  **Intervention/Process:**   - The nurse focuses on the patient within the context of the family and assesses the patient and family using the Friedman Family Assessment Model - Areas assessed include: family stressors, strengths and perceptions; family coping strategies; family adaptation; coping and adaptation over time - The nurse manages the “heart” of the family, not just the “failure” of the patient through helping the family to discovery unique strengths/internal resources and build on them - The nurse informs the patient and family re: patient status and educates them to increase control and decease anxiety   **Other Notes:** Families affect how patients with HF perceive their illness and they also play a significant role in the evolution and outcome of HF and therefore are important to consider in the care plan. |
| Connor, D. (1998). Family-centred care in practice. *Nursing New Zealand (Wellington), 4*(4), 18-19 | Model Development | The “Partnership Care” Approach *(based on the Nottingham Model, by Connor)* | **Pediatric-**especially children with cystic fibrosis and congenital heart disease  **Where:** The pediatric ward at Dunedin Hospital in New Zealand.  **HCP Involved:** Nurses, those who make care plans | **Objectives:**   - To inform parents of what is happening in the care of their child and allow them to be involved in the care to the extent they desire. - To increase desirable outcomes: less frequent admissions, shorter in-patient time, more informed and cooperative parents/careers, greater need for qualified nurses, less need for hospital aids, nurses with improved job satisfaction   **Desired Outcome/End Goal:** Empower patients who want more of a voice in how the care of their child(ren) is delivered in hospital.  **Process/Intervention:**   - Most important change: Care plan is *negotiated* with the family and includes the patient problem and goal of care - Technical jargon is removed from care plans - Negotiation process is documented in care plan - Family and/or child has right to change care plan - Written information is provided to ease anxiety and inform parents of the new approach to care |
| Cormany, E. E. (1993). Family-centered service coordination: a four-tier model. Infants & Young Children: An Interdisciplinary Journal of Early Childhood Intervention, 6(2), 12-19. | Model Development | *Possibly:* A four-tier model of service coordination for young children with special needs and their families. | **Pediatric-**Children with special needs and their families  **Where:** central Florida county and local communities  HCP Involved: Service Coordinators | **Objectives:** Provide a method to improve outcomes for children and their families as they seek services.   - Model objectives are based on assumptions by families, professionals and trends: To improve quality of care, reduce system centered care, recognize child and family concerns, priorities, and resources at the onset of service planning, enable and empower families through promotion of family competencies, participation instead of representation, avoid creating dependence or a learned helplessness by enforcing independent decision making skills, and providing one source for multiple service needs.   **Outcomes/End Goal:** Implementing the required Individualized Family Service Plan (IFSP)  **Process/Intervention:**  Tier 1 – Information and Referral “L.O.V.E line” (Local Outreach for Varying Exceptionalities): entry into simulated information bank by calling a toll-free number. Staff personnel answer questions and gather basic information regarding the child and family.  Tier 2 – Interim Service Coordinator: contacts family within 2 days of call in Tier 1. They conduct an intake meeting to process service inquiry and develop an IFSP.  Tier 3 – Assignment of a Primary Service Coordinator to facilitate future IFSP meetings. The Primary Service Coordinator incorporates the coordination and monitoring of various services (providers, resources and supports)  Tier 4 – Is a subset of the primary service coordination system where the Coordinator’s role becomes that of a typical case manager and service coordinator. |
| Darrah, J., Law, M., & Pollock, N. (2001). Innovations in practice. Family-centered functional therapy -- a choice for children with motor dysfunction. Infants & Young Children: An Interdisciplinary Journal of Early Childhood Intervention, 13(4), 79-87. | Model Development & Evaluation; Feasibility study | Family-centered functional therapy (model of practice) | **Pediatric:** children with motor dysfunction (such as cerebral palsy)  **Where:** rehabilitation centers  **HCP Involved:** Rehab Professionals (OT & PT) | **Objectives:**To help the family and therapist:   1. Promote functional performance by successfully achieving identified functional goals 2. Introduce interventions when a child is trying to accomplish a new task or attempting to do an established task differently (importance of transition) 3. Identify and change primary constraints in the task, child, or environment that are preventing the child from achieving a functional goal 4. Provide opportunities for practice of a new motor skill   **Process/Intervention:**   - Support time-limited, focused treatment that is directed at functional goals identified by the parents and child - Therapists should continue to consider compensatory movement strategies, but also encourage families and children to participate in fitness and play, or sports. - Respect movement choices established by parents and child   **Other Notes:** The attitude change towards FCFT was also influenced by models of disablement, experience of people with disabilities, emerging research, principles of family-centered care and dynamic systems theory. |
| Davison, K. K., Lawson, H. A., & Coatsworth, J. (2012). The Family-Centered Action Model of Intervention Layout and Implementation (FAMILI): The example of childhood obesity. Health Promotion Practice, 13(4), 454-461. doi:http://dx.doi.org/10.1177/1524839910377966 | Model Development | The Family-Centered Action Model of Intervention Layout and Implementation (FAMILI): | **Pediatric-**children with Obesity, but can be used for an array of health problems  **Where:** N/A  **HCP Involved:** nutritionists, etc | **Objective:** To facilitate the design and implementation of research-supported, culturally responsive, and sustainable family-centered programs.    **Desired Outcome/End Goal:** Implement culturally sensitive and sustainable programs to promote healthy family lifestyles.  **Intervention/Process:**  Phase 1: Utilize theories of family development to frame family centered research and practice.   - Objective: Promote the development of research questions that recognize and examine the complexity of interactions within families and the larger systems impacting on family lifestyle behaviors.   Phase 2: Use a mixed-methods approach to examine factors impacting on parents and families that are relevant for intervention design. This process should begin with a qualitative assessment of the realities of daily family life followed by a quantitative assessment of the patterns identified.   - Objective: Facilitate the link between basic and applied research.   Phase 3: Use participatory methods to develop, implement, and evaluate family-centered interventions that empower parents and caregivers to foster healthy family lifestyles and establish systems-level change that reinforces family change.   - Objective: Implement culturally sensitive and sustainable programs to promote healthy family lifestyles.   **Other Notes:** . FAMILI draws on theories of family development to frame research and intervention design, uses a mixed-methods approach to conduct ecologically valid research, and positions family members as active participants in the development, implementation, and evaluation of family-centered obesity prevention programs. |
| Dowling, J., Vender, J., Guilianelli, S., & Wang, B. (2005). A model of family-centered care and satisfaction predictors: the Critical Care Family Assistance Program. Chest, 128(3 Suppl), 81S-92S. doi:https://dx.doi.org/10.1378/chest.128.3_suppl.81S | Model Development & Evaluation; ANOVA analysis | *Possibly:* A model of family-centered care | **Adult:** Patients in the ICU/CCU, and their family members  **Where:** ICU/CCU  **HCP involved:** Nurses, physicians | **Objective(s):**   1. Validate and verify factors that predict patient and family satisfaction in the ICU/CCU 2. Determine whether CCFAP has caused any change in levels of patient and family satisfaction 3. Identify positive and negative factors for any changes   Explore differences in responses between patients and families  **Desired Outcome/End Goal:** Improve patient and family satisfaction in the ICU/CCU  **Process/Intervention:**  Family – computerized survey in waiting room (paper option available)  Patients – surveys mailed to patients post-discharge  **Analyses:**   1. factor analysis and reliability analysis 2. ANOVA 3. standardized mean differences   path analysis  **Other Notes:** Family Members: N=330  (CCFAP Family Satisfaction Survey)  Patients: N=2,266*  (Press-Ganey Inpatient Survey) |
| Feldman, H. M., Ploof, D., & Cohen, W. I. (1999). Physician-family partnerships: the adaptive practice model. Journal of Developmental & Behavioral Pediatrics, 20(2), 111-116. | Model Development | The Adaptive Practice Model | **Pediatric-** children with chronic health conditions and parents.  **Where:** This model can be applied to any clinical setting where a physician interacts with families who have children with chronic health conditions, but not suitable for patients needing chronic care or preventive health maintenance.  **HCP involved:** Physicians | **Objectives:**   - To provide structure for choosing the best approach based on the clinical situation.   **Outcome/End goal:** To increase the likelihood that a family will follow through with the plan because they have been able to participate in decision-making, if a collaborative and supportive style is needed  **Process/Intervention:**   - Four different clinical approaches – directing, teaching, collaborating, and supporting – result from variations in the direction of leadership (physician or family) and in the degree of interaction (high or low). - Sensitive physicians assess the situation and choose an approach based on the needs of the family, re-evaluating and adjusting their approach as needs change  \| High  Degree of  Interaction  Low \| Teaching \| Collaborating \| \| --- \| --- \| --- \| \| Directing \| Supporting \| \| Physician Family  Leadership of the Exchange \| \|      - Interaction refers to the degree of verbal and non-verbal exchange between family and physician - Leadership refers to the participant who exercises primary influence. - *Directing*: suitable when the patient or family has limited knowledge about the situation - *Teaching*: suitable to increase the family’s knowledge and skills - *Collaborating*: families have great degree of knowledge and skill - *Supporting*: families often know more about the condition than the treating physician, but require straightforward instrumental or emotional support - Conflicts can arise if the clinical approach differs from what is expected or desired |
| Gilmer, M. J. (2002). Pediatric palliative care: a family-centered model for critical care. Critical Care Nursing Clinics of North America, 14(2), 207-214. | Model Development; Case Study | Pediatric Palliative Care: A Family-centred Model for Critical Care | **Parents/Pediatric-** This model targets families caring for critically ill children receiving palliative care.  **Where:** Hospitals, medical centers, hospices, home (not clearly specified)  **HCP involved**: An interdisciplinary team of nurses, physicians, social workers, chaplains, child life specialists, pharmacists, ethicists, bereavement counselors, and volunteers | **Objectives:**   - Critical and palliative care share similar essential elements and goals - Goal of care should be an appropriate balance between palliative and critical care   **End goal/Desired Outcome:** Add life to a child’s time rather than just time to a child’s life  **Process/Intervention:** The model considers the characteristics of the family and the child and how these features impact their needs. The model addresses the physical, mental (including emotional and social), and spiritual needs of families through the following:   1. Clinical services – involving family in a family-centered plan of care at time of diagnosis, and further communicating with them about treatment options and decision-making 2. Education and training of doctors and nurses to ensure adequate knowledge and skill in providing palliative care to children 3. Support services – support groups for parents and siblings, retreat services, respite care, and bereavement support   **Other notes:** Implementing a family-centered approach encourages families’ participation in a collaborative and supportive partnership and facilitates seamless continuity in addressing children’s and families’ needs related to life-threatening conditions. A family’s needs are addressed when health care professionals tend to the physical needs of the child. |
| Goetz, D. R., & Caron, W. (1999). A biopsychosocial model for youth obesity: consideration of an ecosystemic collaboration. International Journal of Obesity & Related Metabolic Disorders: Journal of the International Association for the Study of Obesity, 23 Suppl 2, S58-64. | Model Development | The Family-Collaborative Ecosystemic Model (FEM) | **Pediatric:** obese youth and their families.  **Where:** This model can be implemented in any health facility treating youth with obesity, where collaboration between medical and psychosocial professions is possible.  **HCP involved:** Social workers, doctors, psychologists, nutritionists, exercise physiologists, movement therapists, etc. | **Objectives:** (not explicitly stated)   - To promote balance, harmony, and healing for the obese individual using a holistic framework. - To promote collaboration and help health care professionals go beyond the disease concept by avoiding the stigmatization of obesity. - To shift from a pathological perspective to a strengths perspective.   **End Goal:** Improve the care for obese youth  **Intervention/Process:** Objectives are achieved through the Ecosystemic Biopsychosocial Grid (EBG), a clinically useful tool to be used by a variety of healthcare professionals. Obesity is viewed as a failed attempt of achieving harmony and balance in the patient’s psychological world and in interactions their family and society.   1. Goals are established by a collaborative discussion among health care professionals, the patient and the patient’s family. 2. The EBG is used to identify various factors related to achieving the goal, such as existential/spiritual, cultural, social, family, psychological, biomedical, etc. Professionals take on various domains as appropriate. 3. Obstacles (unbalance), Resources (balance), Solutions, and Targets are determined. 4. Interventions arise out of using the EBG, and should focus on enhancing the influence of resources.   Other Notes: A diverse approach that integrates family systems theory, ecosystems theory, and biopsychosocial theory. The FEM also integrates Eastern and Western views of health. |
| Grebin, B., Kaplan, S. C., & Zimmer, E. P. (1995). Toward a pediatric subacute care model: Clinical and administrative features. Archives of Physical Medicine and Rehabilitation, 76(12 SUPPL.), SC16-SC20. doi:http://dx.doi.org/10.1016/S0003-9993%2895%2981398-5 | Model Development; Case Study | Pediatric Subacute Care Model | **Pediatric/Parents:** This model targets the families of children being treated at the subacute level  **Where:** Subacute care is a level of care defined by certain distinct features, rather than one specific place, and therefore takes place across a variety of settings:   - Inpatient - Home - Medical daycare   **HCP Involved:** A specialized team of staff should be specifically recruited and hired to meet the medical, psychological, social, and educational needs of children – pediatricians, pediatric nurses, child psychologists and psychiatrists, therapists from various disciplines, home care staff, social workers, early interventionists, early education teachers and assistants | **Objectives:**   - Significant reduction in long-term acute care pediatric patient stays by having additional beds available for subacute care - Family involvement is the key to a child’s return home   Desired Outcome/End Goal: increase cost-effectiveness and age-appropriateness.  **Process/Intervention:**   - Goal-oriented treatment after, or instead of, acute hospitalization to treat complex medical conditions or administer complex medical treatments - Overall training, education, and support of family - Opportunities for family to be part of everyday care – participation with medical team, helping in therapy, feeding - Training in administering medications and changing bandages - Creation of a physical environment that is tailored to meet the needs of children - Linking families to community services |
| Hernandez, L. P., & Lucero, E. (1996). Days La Familia community drug and alcohol prevention program: Family-centered model for working with inner-city Hispanic families. Journal of Primary Prevention, 16(3), 255-272. doi:https://dx.doi.org/10.1007/BF02407425 | Model Development & Evaluation; Pre-test, post-test design. | DAYS La familia community drug and alcohol prevention program: Family-centered model | **Families with High-Risk Youth-** Hispanic families with high-risk youth from 6-11 years old.  **Where:** An inner-city American community with high risk of substance abuse among its youth.  **HCP involved:** Human services staff i.e. Social workers | **Objectives:** To build on existing family, community and individual resources to protect people from risk factors including: family disruption and disunity, mental health problems, deviant behaviour, negative peer influences, early use of drugs, and strong and favourable attitudes towards the use of alcohol and drugs.  **End Goal/Desired Outcome:** Drug prevention  **Intervention/Process:**  There are 8 defining tasks of La Familia.   1. Utilize existing community relationships, networks and leadership for outreach and recruitment 2. Let every person identify his or her own priorities and goals 3. Help build trusting relationships between family members and other families 4. Teach parents and other community members to be “prevention minded” 5. Help children, parents, families, and communities implement new skills and focus on “doable” and “winnable” target behaviours and achieve small victories 6. Teach parents and other community members to organize and build supportive relationships with groups and individuals that have resources and willingness to help them achieve their goals 7. Train as many community-based prevention specialists, parent organizers, and peer counselors as possible 8. Encourage greater independent action of child and families early to avoid dependence on the program   The program has three components:   1. Family Strengths Program (FSP): 14 weeks to address risk factors and focus on parenting skills, a positive family environment, communication, children’s social skills, etc. 2. Basic Prevention Program (BPP): emphasis on applied prevention and intervention principles. Explores drug use attitudes and awareness, family bonding, self-esteem, personal efficacy, developing positive peer influences and responsible behaviour in community. 3. Follow-Along Program (FAP): Reinforces what has been learned in the other components, and links participants with community resources, and positive peer and adult influences. |
| Heuer, S. (2007). Family-centered care. Integrated medical and dental health in primary care. *Journal for Specialists in Pediatric Nursing, 12*(1), 61-65. | Model Development | *Possibly:* Neighborhood Outreach Action for Health (NOAH) family-centered model of integrated service delivery | **Pediatric:** children and parents of low-income, uninsured families with suffer from oral disease  **Where:** school-based and school-linked health centers providing primary medical care  **HCP Involved:** Dental hygienist, nurse practitioners | **Objectives(s):**   - To integrate service delivery - To improve the health of low-income, uninsured children who suffer disproportionately from oral disease   **Process/Intervention**   - Placing a greater emphasis on oral screenings and oral health education - Teaching mothers how to examine their infant’s mouth for gum disease - Discussing age-appropriate nutrition - Showing oral health videos   **Other Notes:** Tooth decay can affect health in adulthood. Data emerged that supported the positive impact of oral health on overall well-being. Nurse practitioners believed that the integration of families in comprehensive care would improve oral health/hygiene education and maintain oral health post restorative interventions. |
| Hyman, D. (2003).Reorganizing health systems to promote best practice medical care, patient self-management, and family-centered care for childhood asthma. *Ethnicity & Disease, 13*(3 Suppl 3), S3-94-98.) | Model Development | Care Model for Child Health | **Pediatric :** Children diagnosed with, or are suffering from symptoms of asthma and their families  **Where:**  **HCP Involved:** physicians | **Objectives:** Improve functional and clinical outcomes for children with asthma by creating a partnership between motivated patients, families, and proactive practice teams.  Desired Outcome: Improve the care gap in asthma  **Process/Intervention:** By taking into account 6 care components:   1. Decision support: supporting care decisions with evidence based principles, agreeing upon guidelines 2. Delivery system design: making reliable care processes by changing office systems and rethinking who is responsible for each step of these processes 3. Clinical information systems: using electronic (or paper) databases and registries to identify patients in need of services, allow care at regular planned intervals depending on disease status 4. Family and self-management support: focus is on how asthma interferes with everyday life. A primary tenet is to actively involve family and patient in managing the disease. Steps to follow are (a) Establish a Focus: learn from the patient and family about their chief issues and concerns about the disease; (b) Share Information: allow patient and family to make informed decision about where to focus their efforts by sharing disease information and emphasizing concerns; (c) Develop a Shared Goal: collaborating by incorporating HCP’s and family’s perspective to set goals but to make these achievable by breaking them down to smaller steps; (d) Develop an Action Plan: discuss the how, what, when, where, and frequency of the new goal/behavior as well as possible barriers to success; (e) Use Problem-Solving Techniques: use problem solving methods to overcome barriers and problems identified during action planning; (f) Follow-up: agreeing with the patient and family with a follow-up plan to show that the patient and family will be expected to report on progress towards the established goal. 5. Community resources: ensure patient and family are linked to community resources 6. Health care organizations: work towards a regulatory payment and policies systems that facilitates optimal care.   **Other Notes:** There are shortfalls in the management of children with asthma recently demonstrated by a series of published studies. A care gap exists that needs to be recognized and addressed to improve asthma care. A 1997 report states that asthma symptoms should almost always be controlled. However, epidemiological data shows otherwise. Acute conditions are better managed than chronic conditions due to lack of training in care coordination. Key components of asthma care are use of inhaled corticosteroids, written treatment plans and review of environmental tobacco smoke exposure. There needs to be a reliable provision of these components of asthma care. |
| Jasovsky, D. A., Morrow, M. R., Clementi, P. S., & Hindle, P. A. (2010). Theories in action and how nursing practice changed. *Nursing Science Quarterly, 23*(1), 29-38. doi:https://dx.doi.org/10.1177/0894318409353806 | Model Development; Literature Review | Magis Model of Care – A collaborative approach to care-giving and decision making with patient/family and all members providing knowledge, skills, and experience in each healthcare encounter. | **Who:** N/A  **Where:** Cardiovascular unit of a Chicago hospital  **HCP involved:** Primarily nursing; social workers, physical therapists, dieticians, pastoral care and respiratory practitioners | **Objectives:** (goal) Treat the human spirit by caring for patient/family members using Magis value-driven service with each encounter  **Process/Intervention:** 7 tiers  Presence (behaviors), purpose, and practice outcomes of care, concern, cooperation, and respect with the patient/family at the center of care.  Examples:   - Purpose of care: Patient/family encouraged and supported in participating in care and decision-making at the level they choose - Purpose of concern: Healthcare practitioners communicate and share unbiased information with patient/family in ways that are affirming and useful. Patient/family receive timely, complete, and accurate information in order to effectively participate in care and decision-making at the level they choose. - Purpose of cooperation: Patient/family are included at the institution level and collaborate in policy and program development, implementation, and evaluation of facility design, professional education, and delivery of care. - Purpose of respect: Listen and honor patient/family knowledge, values, beliefs, and cultural background part of care plan and delivery of care.   **Other Notes:** To receive the Magnet designation from the American Nurses Credentialing Center, the Chief Nursing Officer (CNO) must complete the first component of the designation, which is transformational leadership (TL). In order to do so, the CNO must have a strong vision, philosophy, practice model, and both strategic and quality plans to lead nursing services. |
| Kaufman, J. (1992). Case management services for children with special health care needs. A family-centered approach. Journal of Case Management, 1(2), 53-56. | Model Development; Case Study | *Possibly:* A Family-Centered Approach to Case Management Services for Children with Special Health Care Needs | **Pediatric:** This model targets children with complex medical needs and their families.  **Where:** This model is implemented in the transition from hospital to home by the Coordinating Centre for Home and Community Care (CCHCC) – a private, non-profit community based organization - in Maryland, USA.  **HCP Involved:** Case Managers | **Objectives:**   - To locate, coordinate and monitor the quality, continuity, provision and cost of services at home. - To provide a cost-effective way to meet the optical medical, educational and psychosocial needs of the child and family. - To enable the organization to make independent, educating decisions regarding children’s health care needs, eliminating need for professional case manager. - To enable families to eventually become the case managers for their children.   **Desired Outcome/End Goal:** This model allows children to move into less costly care facilities or home – a more humane choice for families and a reduction in cost for funders.  **Process/Intervention:**   - Educate and information-sharing with parents and encourage them to make cost-conscious choices and decrease the need of ongoing professional support - Planning - pre-discharge: hospital personnel train family members in the child’s health care needs, conduct a needs assessment with the family at home, identify appropriate providers of care based on level of need, analyze the costs associated with home care, and arrange for all education-related services - Implementation: -a pre-discharge meeting including all relevant stakeholders to develop a detailed initial plan of care (POC), teaching family about emergency situations and re-admission protocol, reviewing of financial parameters, and continuous follow-up and POC monitoring by the CCHCC staff - On-site and telephone contact with family, home care providers, and funders on a regular basis - Termination of case management services may occur if family is able to become their own case managers or if the child no longer requires specialized home care services |
| Kavanagh, K. T., & Tate, N. P. (1990). Models to promote medical health care delivery for indigent families: computerized tracking to case management. Journal of Health & Social Policy, 2(1), 21-34. doi:https://dx.doi.org/10.1300/J045v02n01_03 | Model Development | Family-centered case management (FCCM) | **Pediatric:** children and parents of indigent families with poor medical compliance  **Where:** tightly woven communities resistant to outside intervention  **HCP Involved:** physician, healthcare workers, social workers | **Objectives:**   - To promote patient compliance - To coordinate medical and social services - To support the family   **End Goal/Desired Outcome:** get families out of poverty, and aid to enter the marketplace  **Process/Intervention:**   - Providing case management that supports the family and coordinates their medical and social services. - Best suited to provide care to medically complex families (multiple-handicapped children)   **Other Notes:** Simply providing the availability of medical care is insufficient. Neither child nor society has the obligation to ensure that children obtain needed care. This responsibility falls on the physician or healthcare worker. However, complex socioeconomic factors result in significantly lower appointment attendance rates. Most simple medical issues become complex ones. Family and parental factors must be addressed to increase compliance rates. |
| Kazak, A. E. (2001). Comprehensive care for children with cancer and their families: A social ecological framework guiding research, practice, and policy. *Children's Services: Social Policy, Research, & Practice, 4*(4), 217-233. doi:http://dx.doi.org/10.1207/S15326918CS0404_05 | Model Development | Surviving Cancer Competently Intervention Program (SCCIP) - Treatment model for cancer survival | **Pediatric-**Children with cancer and their families  **Where:** children’s hospital, oncology department  **HCP involved**: nurses, social workers, and psychologists | **Objectives:** To lower anxiety and posttraumatic stress in adolescent childhood cancer survivors, their parents and siblings.  **Desired Outcome:** Less anxiety and posttraumatic stress  **Intervention/Process:**  The SCCIP integrates cognitive behavioral and family therapy approaches,  drawing heavily on adaptations of the Adversities–Beliefs–Consequences  (A–B–C) Model (Seligman, 1990), and multiple family discussion groups approaches (Steinglass, 1998). The SCCIP consists of four sessions within 1  (weekend) day for a group of families. During the two morning sessions, separate  groups (of survivors, mothers, fathers, siblings) identify beliefs about cancer  and its treatment and learn and practice the A–B–C Model and reframing. In the  afternoon, two sessions link individual’s beliefs to how these beliefs affect the  family and promote or hinder ongoing developmental growth for the children  and families.  **Other Notes:** Studies indicate that preventive interventions might reduce the likelihood of later PTS by reducing parental distress and enhancing self-efficacy during treatment; 3) Data indicates that higher levels of symptoms in young adult survivors provide support for interventions during adolescence and young adults since symptoms of posttraumatic stress could promote reluctance (or even outright avoidance) of medical care. |
| King, G., Tucker, M. A., Baldwin, P., Lowry, K., LaPorta, J., & Martens, L. (2002). A life needs model of pediatric service delivery: services to support community participation and quality of life for children and youth with disabilities. Physical & Occupational Therapy in Pediatrics, 22(2), 53-77. | Model Development; Literature Review | Life Needs Model of Pediatric Service Delivery (an applied developmental and socio-ecological model that focuses on structure (services structured to meet needs, process (family-centered services), and outcomes (participation and quality of life) | **Pediatric-** This model applies to children involved in pediatric rehabilitation and their families at key transitions during the course of the child’s life.  **Where:** This model was developed by the Thames Valley Children’s Centre in a Canadian context but has flexibility to be applied to other regions and populations.  **HCP Involved:** A transdisciplinary service delivery model so that there is a holistic, common goal and way of viewing services among rehabilitation science professionals, social workers, psychologists, case managers and researchers in the areas of early intervention and developmental disability | **Objectives:**   - To support children’s participation in all areas of life. When needs are met, opportunities are maximized in areas important to children therefore contributing to quality of life. - To outline the scope of major types of age-specific service delivery needs for children and their families in a geographic region in order to meet long-range goals of community participation and quality of life - To emphasize the importance of meeting many needs, not just the individualized therapy needs of the child   **Desired Outcome/End Goal:** Children thrive in their communities, rather than simply function  **Process/Intervention:**  Service Delivery Themes:   1. The importance of focusing on strengths of children and families (i.e. Empowerment and resilience) 2. The need to focus on children’s real-world function and participation in addition to intervening on the level of impairment 3. The importance of providing service options at transition points 4. The importance of fostering nurturing and accepting environments to support community participation and quality of life  - Child, family and community are all in the foreground as simultaneous stakeholders - Outlines short-term goals of services in the external sphere, interpersonal sphere and personal sphere of life - Defines the needed services in these spheres:   - External sphere: Services addressing community needs for information and education   - Interpersonal sphere:     - Services addressing parents’ and family members’ needs for support, information and skill development     - Services addressing clients’ needs for support and information     - Services focusing on clients’ applied skill sets   - Personal sphere: Services focusing on client’s foundational skills (to meet physical, social, emotional, communication and behavioural needs) - Interpersonal sphere: where family members’ needs are addressed   - In addition to healthcare providers recognizing and responding to families’ needs in informal ways, a variety of programs were created to address parents’ needs for information, education and skill development   - Based on the premises that people are experts in their own situation and function best in a supportive family/community environment, services should be provided in a family-centered way. This means:     - Clients and families should decide their level of involvement in decision-making     - Children and families should be treated with respect     - Needs of all family members should be considered     - Involvement of all family members should be supported and encouraged (Rosenbaum et al., 1998)   **Other Notes:** Communities do not have an adequate service delivery framework (as opposed to a conceptual framework) to address the spectrum of needs of children and youth with disabilities and their families. The Life Needs Model also takes into account the developmental status of the community with respect to attitudes, policies, and practices that support inclusion. |
| Kissane, D. (2003). Family focused grief therapy: the role of the family in preventive and therapeutic bereavement care. Bereavement Care, 22(1), 6-8. | Model Development; RCT | Family focused grief therapy (FFGT) | **Palliative/Adult:** This model targets patients and families in end-of-life care and families during bereavement  **Where:** Hospice Care  **HCP Involved:** Therapist | **Objectives:** To enhance the functioning of the family by exploring their cohesiveness, communication of thoughts and feelings, and resolution of conflict.  **Desired Outcome/End Goal:** Cope with grief  **Process/Intervention:** is brief, focused and time-limited, usually extending flexibly  across 6 to 18 months. Each therapy session lasts 90 minutes, and the whole course of 4 - 8 sessions can be divided into three sequential phases:   1. Assessment (one/two weekly sessions) involving identification of relevant issues or concerns for the family and negotiation of a therapeutic plan to work on these; 2. Intervention (typically two/four fortnightly or monthly sessions), focusing on the agreed concerns; 3. Termination (one or two sessions at two- or three-month intervals) consolidating and ending the therapy. The frequency and total number of sessions are adapted to each family’s context and needs. Although developed for the families of adult cancer sufferers, adolescents, children and grandchildren automatically become involved in FFGT, so this seems to be a model that can be generalised.   Training for therapists is very important so that as facilitators they can remain neutral amid the varied opinions, generate hypotheses about relationship issues, and ask questions in both a circular manner to collect data and strategically to guide members to a constructive resolution of problems2  **Other Notes:** Developed within hospice care as an empirically derived model targeting the family as the most naturally occurring social group for the bereaved  A recent controlled trial comparing bereavement care in a palliative setting with that in a conventional hospital failed to demonstrate any improvement in bereavement outcome. This highlights the challenge for the hospice to successfully integrate family care into its practices. FFGT offers an approach that allows supportive therapy to begin before the patient's death and sustains continuity of care throughout bereavement. |
| Kissane, D., Lichtenthal, W. G., & Zaider, T. (2008). Family care before and after bereavement. Omega: Journal of Death and Dying, 56(1), 21-32. doi:http://dx.doi.org/10.2190/OM.56.1.c | Model Development; RCT | The Family Focused Grief Therapy (FFGT) Model | **Palliative/Adult:** This model targets patients and families in end-of-life care and families during bereavement.  **Where:** This model was developed and evaluated at Memorial Sloan-Ketting Cancer Center in New York. It can be adopted for use in any palliative care setting.  **HCP Involved;** Therapists, Psychologists | **Objectives:**   - To decrease the distress of families during end-of-life care and after the patient has died. - To optimize cohesion, communication of thoughts and feelings, and the handling of conflict while promoting the sharing of grief and mutual support.   **Desired Outcome/End Goal:** To prevent or ameliorate the suffering associated with various forms of pathological grief.    **Process/Intervention:** The Family Relationships Index (FRI) is administered to families to determine members’ perception of family’s cohesiveness, expressiveness, and capacity to deal with conflict. Based on the outcome of the scale, families are identified as at-risk for psychosocial morbidity and declining function under the strain of death and bereavement.  Family therapy:   - Focused and time-limited (6-10 sessions across 9-18 months) - Three phases: assessment, intervention (focuses on agreed concerns), and termination (gains are consolidated) - Concentrating on family-as-a-whole - Interventions encourage family empathy - Balances modifications of family’s shared values, ideas, attitudes, and beliefs with preservation of their relational identity - Strengths-based – harness areas of competence, affirm skills, and identify capacities for growth   Instruments:  Measures of family functioning   - Family Environment Scale, short form (FES) - Family Assessment Device (FAD)   Measure of distress   - Brief Symptom Inventory (BSI) and it’s General Severity Index (BSI-GSI)   Measure of depression   - Beck Depression Inventory, short form (BDI)   Measure of social adjustment   - Social Adjustment Scale (SAS)   Measure of grief   - Bereavement Phenomenology Questionnaire (BPQ)   **Analyses:**  - Generalized estimating equations (GEE)  - Identity link function + Huber/White robust sandwich variance estimates with an independent covariance matrix  **Other Notes:** The FFGT is a preventive model based on Cicely Saunders of St. Joseph’s’ idea. It was developed in the absence of well-developed approaches to family grief.  **Significant Findings:**   - N=40 families who completed treatment - Significant reduction in distress after 13 months of bereavement. There was little impact on social adjustment or family functioning. - Significant reduction in both distress and depression for the 10% of families who were most distressed at baseline. There was a trend towards improved social functioning. - Effect size for intermediate functioning families was large (0.37), small (0.16) for sullen families, and medium (0.29) for hostile families. - Program completers reported that the treatment was significantly more helpful than noncompleters |
| Law, M., Darrah, J., Pollock, N., King, G., Rosenbaum, P., Russell, D., . . . Watt, J. (1998). Family-centred functional therapy for children with cerebral palsy: An emerging practice model. Physical and Occupational Therapy in Pediatrics, 18(1), 83-102. | Model Development & Evaluation; Feasibility study | Family-centered functional therapy (FCFT) – an emerging practice model | **Pediatric:** Children with cerebral palsy and their families  **Where:** children’s rehabilitation centers  **HCP involved:** Rehab. professionals (physical and occupational therapy) | **Objectives:**   - To identify and change performance constraints in the task, the child, or the environment   **Desired Outcome/End Goal:** improved function and motor-based activities  **Process/Intervention:**   1. Promote functional performance 2. Identify periods of change 3. Identify and change the primary constraints in the task, child and/or environment that prevent achievement of the task 4. Encourage practice   **Other Notes:** Neurodevelopment treatment (NDT) was often used as therapy for children with cerebral palsy. However, lack of evidence supporting its efficacy had therapists considering other movement solutions. Functional abilities of children with CP should be viewed by concepts emerging from a systems approach in motor development and a family-centered approach. |
| Leviton, A., Mueller, M., & Kauffman, C. (1992). The family-centered consultation model: practical applications for professionals. Infants & Young Children: An Interdisciplinary Journal of Early Childhood Intervention, 4(3), 1-8. | Model Development | The family-centered consultation model | **Pediatric:** Children with special needs (developmentally disabled or at risk) and their families  **Where:** unspecified - service delivery  **HCP involved:** not specified, but should also include policy making, advocacy | **Objectives:** To assist professionals and agency administrators in implementing family centered care practices (comparison is made between the professional's role in traditional models of intervention and his or her role in the family-centered consultation model of intervention).  **Desired Outcome/End Goal:** Improved family centered care practices  **Process/Intervention:**  Stage 1: Determining the parent-professional relationship and goals   - Parent chooses the role as well as the degree of involvement of the professional - Both parties jointly provide information to assist in making informed decisions about services and resources the family would like to receive   Stage 2: Generating strategies to achieve goals   - Family and professional meet to discuss a variety of options in order to reach the family’s goals. These are based on their interests and concerns. The professional presents the options while avoiding recommendations. Thus, strategies are formulated to achieve maximum benefit for child and family.   Stage 3: Decision making and service delivery   - Once the plan in decided, parents begin receiving services from the professional as needed. Roles may start to vary and needs may change. Families may request a wide range of services from the professional.   Practical suggestions for implementation in four service delivery areas: referral, assessment, paperwork and meetings.  **Other notes**: The rationale behind this model was, Legislation like part H of PL 99-457 necessitates the development of Individualized Family Service Plans (IFSP), and thus requires states to make family-centered care a reality. |
| Lyon, M. E., Garvie, P. A., Briggs, L., He, J., McCarter, R., & D'Angelo, L. J. (2009). Development, feasibility, and acceptability of the Family/Adolescent-Centered (FACE) Advance Care Planning intervention for adolescents with HIV. *Journal of Palliative Medicine, 12*(4), 363-372. doi:https://dx.doi.org/10.1089/jpm.2008.0261 | Model Development; RCT | the Family/Adolescent-Centered (FACE) Advance Care Planning intervention | **Pediatric/Adolsences-** with HIV and their families  Where:  HCP Involved; | **Objectives:** to test the efficacy of FAmily CEntered Advance Care Planning among adults living with AIDS and/or HIV with co-morbidities on congruence in treatment preferences, healthcare utilization, and quality of life. The FAmily CEntered intervention arm is two face-to-face sessions with a trained, certified facilitator:  **End goal/Desired Outcome:**  that this intervention will enhance patient-centered communication with a surrogate decision-maker about end of life treatment preferences over time, enhance patient quality of life and decrease health care utilization.  **Process/Intervention:** two face-to-face sessions with a trained, certified facilitator: Session 1) Disease-Specific Advance Care Planning Respecting Choices Interview; Session 2) Completion of advance directive. The Healthy Living control arm is: Session 1) Developmental/Relationship History; Session 2) Nutrition.  **Other notes:** Follow-up data will be collected at 3, 6, 12, and 18-month post-intervention. A total of 288 patient/surrogate dyads will be enrolled from five hospital-based, out-patient clinics in Washington, District of Columbia. Participants will be HIV positive and ≥21 years of age; surrogates will be ≥18 years of age. Exclusion criteria are homicidality, suicidality, psychosis, and impaired cognitive functioning. |
| MacFarlane, M. M. (2011). Family centered care in adult mental health: Developing a collaborative interagency practice. Journal of Family Psychotherapy, 22(1), 56-73. doi:http://dx.doi.org/10.1080/08975353.2011.551100 | Model Development; Literature Review | Family Centered Care Working Group (FCCWG) | **Adult:** adult mental health patients and their families  **Where:** Ross Memorial Hospital IP, day treatment, and outpatient programs  **FCC Involved:** psychiatrists, psychiatric nurses,  social workers, family practitioners, a hospitalist, and a marriage and family  therapist | **Objectives:**   1. To better meet the needs of family members of patients’ with a mental health illness 2. To obtain as broad a cross-section of input into family needs and issues as possible 3. To encourage as broad a dissemination of information as possible 4. To identify gaps in services to families coping with mental illness and ways to address these gaps   **Intervention/Process:**   1. Creating a family-centered care working group 2. Family focus groups 3. Actions to address the needs identified by points 1&2 above  - establish a designated contact person for family members in the IP unit - one-page information sheet for family members of IPs was created for staff to give to family when their loved one is admitted or at the family member’s first visit (relevant phone numbers, contact persons, the purpose of the IP unit as acute care and stabilization vs. intensive or long-term (for clear understanding of what to expect from treatment), visiting hours, items family members may or should not bring for their loved one). - “Mental Health Supports for Families” sheet with information on family support services offered with phone numbers for contacting each organization - establishment of a resource library at the hospital for written information about various mental illnesses - video library of educational films describing mental health issues and their impact on the family   “Journeying Together Family Support Program”: a mix of group supports and brief services to individual families. There are 3 structured sessions to provide a psychosocial assessment for the caregiver, an assessment of caregiver strengths, information on self-care, instruction in communication and problem solving, and information and education regarding the mental illness the family member’s loved one is experiencing including educational information regarding medication and treatment options. |
| Madigan, C. K., Donaghue, D. D., & Carpenter, E. V. (1999). Development of a family liaison model during operative procedures. MCN, American Journal of Maternal Child Nursing, 24(4), 185-189. | Model Development; Case Study | Family Liaison Model | **Pediatric-**  patients and their families in cardiac intensive care units (CICU) during operative procedures.  **Where:** This model was developed in the Cardiac Intensive Care Unit at The Children’s Hospital of Philadelphia.  **HCP involved**: Nursing staff of the CICU | **Objectives:**   - To facilitate the establishment of a relationship between CICU nursing staff, parents and families at the earliest possible point in time - To ensure communication with parents and families at regular intervals during their child’s surgery - To promote practice that incorporates principles of family-centred care with the CICU (such as respect for cultural diversity, acknowledgement of strengths, exchanging unbiased information, encouraging family-to-family support, appreciating families as families, and ensuring that support systems are accessible and comprehensive)   **End Goal/Desired Outcome:** Increase satisfaction of parents due to lack of parent-nurse communication.  **Process/Intervention:**   - Educate parents and families - Communicate updates during preoperative, intra-operative, and post-operative stages of the procedure   - Pre-op: be present for family in holding area when anesthesiologist arrives   - Intra-op: Bring them to parent lounge, review what to expect, and give periodic updates to parents every 45-60 minutes and answer questions   - Post-op: Ensure family can see their child within 45 minutes after procedure - Provide physical and emotional support - Establish continuity of care for the patient and family immediately after operative procedure - Nurses received additional training on heart surgery procedures, time frames so that they were better able to relate this information to parents and families - Nurses also became better acquainted with additional services such as social workers and bereavement coordinator   **Other notes:** This model was developed in response to reduced satisfaction of parents due to lack of parent-nurse communication. |
| Marcenko, M. O., & Smith, L. K. (1992). The impact of a family-centered case management approach. Social Work in Health Care, 17(1), 87-100. doi:https://dx.doi.org/10.1300/J010v17n01_06 | Model Evaluation; Mixed Methods | *Possibly:* Family-Centered Case Management Model | **Pediatric:** This model targets children with developmental disabilities and their families.  **Where:** Urban Michigan communities; across the care continuum to coordinate a range of programs, services, and supports  **HCP involved:** Social workers in collaboration with nurses, doctors and teachers as appropriate | **Objectives:** Assure service accessibility, continuity, quality of care, and responsiveness  **End goal/desired outcome:** Maximize potential of persons with disabilities and their families for independence, productivity, and community integration  **Intervention/Process:**  Functions:   - Outreach - Coordination - Brokering - Monitoring - Advocating - Training - Interdisciplinary team planning   Major activities include:   - Working with parent groups - Arranging support services - Obtaining financing for services - Counseling - Administration   - All needs of family members are taken into account  - Mutually supportive relationship with families (not adversarial)  - Increased professional availability to accommodate busy family schedules  - Empowerment of families through support groups, information sessions, and skill building sessions |
| Martin-Arafeh, J. M., Watson, C. L., & Baird, S. M. (1999). Promoting family-centered care in high risk pregnancy. Journal of Perinatal & Neonatal Nursing, 13(1), 27-42; quiz 94-25. | Model Development; Literature Review | Calgary Family Assessment Model/Calgary Family Intervention Model | **Maternal Health:** This model targets women experience high-risk pregnancies and their families. It can be applied to any population in a healthcare setting.  **Where:** Hospital setting. Implemented at Foothills Hospital in Calgary, Canada  **HCP involved:** Nuses, clinical nurse specialist | **Objectives:** To meet the needs of the family through thorough assessment and implementation of strategies to promote family support  **Desired outcome/End goal:** Needs will be better met through assessment of the family and interventions to support the family.  **Process/Intervention:**  Elements of Family Centered Care   1. Family as the constant 2. Collaboration: health care professionals need to support and educate the family by sharing information at an understandable level in order to enable shared decision making 3. Respect the uniqueness of families 4. Families supporting families through friendship, peer support, and sharing of information and resources   CFAM: Calgary Family Assessment Model  Structural assessment – attachment of family members  Developmental assessment – examines significant events, stages, perceptions  Functional assessment – how members interact  CFIM: Calgary Family Implementation Model  How to cause change to promote family support for each other  CFIM Strategies:   - Commending strengths - Providing education - Validating emotions - Allowing them to share the experience of the illness - Encouraging family support - Encouraging members to be involved in patient care - Encouraging respite - Maintaining family rituals   Nurses require a non-threatening environment to implement family centered care. Hospitals must establish policies and programs that are aligned with family needs. |
| Mausner, S. (1995). Families helping families: an innovative approach to the provision of respite care for families of children with complex medical needs. Social Work in Health Care, 21(1), 95-106. doi:https://dx.doi.org/10.1300/J010v21n01_08 | Model Development | Families Helping Families | **Pediatric Families:** families of children with complex medical needs  **Where:** private family homes  **HCP Involved:** social workers, nurses (community health) | **Objectives:** To provide respite care to parents of children with complex medical needs  **Process/Intervention:** Through the location of provider families by the social worker, training of provider families by the project nurse, and support for the families through a parent liaison with a medically involved child. Family issues that arose are communicated to the rest of the team by the parent liaison.  **Other Notes:** professionals could be provided by an appropriate “sharing” family. |
| McKlindon, D., & Barnsteiner, J. H. (1999). Therapeutic relationships. Evolution of the Children's Hospital of Philadelphia model. MCN, American Journal of Maternal Child Nursing, 24(5), 237-243. | Model Development | Children’s Hospital of Philadelphia (CHOP) Model | **Pediatric:** as well as the health care staff that work with them  **Where:** Pediatric care facilities (type and level of care not specified)  **HCP Involved:** Pediatric nursing | **Objectives:**   - To support health care needs of families by establishing partnerships between families and health care professionals that span the health care continuum - “Therapeutic Relationship” as standard of practice – to establish and maintain relationships between nursing staff and families that are caring, clear, boundaried, positive, and professional   **End Goal/Desired Outcome:** Better supported family caregivers  **Process/Intervention:**   1. Empowerment of families to maintain control over their own decision making and care 2. Management of interpersonal boundaries, which includes:    1. Boundaries between staff and families: balance personal and professional, monitor self-disclosure, identify family strengths, treat all families and family members equally    2. The delivery of care and the care delivery system: assist parents in understanding the system and provide care and resources based on needs    3. Closure and transition of care: direct communication, fostering a sense of trust 3. Support families in their care-giving role, promote them as integral members of the care team, and encourage them to utilize available supports |
| Monahan, D. J. (1993). Assessment of dementia patients and their families: an ecological-family-centered approach. Health & Social Work, 18(2), 123-131. | Model Development; Case Study | *Possibly:* Ecological-Family-Centered Approach | **Adult:** In this article, the model is used to guide assessment of patients with dementia and their families.  **Where:** Dementia Evaluation Clinic affiliated with area university hospital  **HCP involved:** Multidisciplinary healthcare team: social workers, nurses and physicians | **Objectives:**   - To select ethnically sensitive intervention strategies - To enable the social worker to examine physiological, environmental, cultural factors and living arrangements of the client and family - To improve assessment, diagnoses, and treatment through input of family members/caregivers - To help social workers determine the viability of interventions based on challenges and strengths of family and client assessment   **End Goal/Desired Outcome:** Guide the assessment of persons with dementia  **Process/Intervention:** Using an ecological framework to systematically analyze the situation. Consists of seven variables: four assessment variables, which are used to help evaluate the usefulness of three intervention variables, for both caregivers and dementia patients.  Assessment variables:   1. Physiological factors 2. Environmental or situational factors 3. Culturally based conflicts 4. Living arrangements   Intervention variables:   1. Can educational or skills training help? 2. Can group experiences help? 3. Can individual or family counseling help?   Conceptualized as: P+E+C+L 🡪 ES, G, IFC   - Pre-questionnaire re: demographic data, person with dementia’s physical functioning, family’s knowledge and use of resources, and caregiver stress - Multidisciplinary health-care team reviews questionnaire, screens patient and meets with family   Over next few visits:   - Complete medical assessment and formulation of diagnosis, potential medication prescription - Psychosocial evaluation of family members and patient re: assessment variables – intervene if necessary: counseling, group experiences, education or skills training - (For approximately 8 weeks) Continue to monitor effects of psychosocial interventions and medication   **Other Notes:** In the context of dementia, family members play an integral caregiving role and require support for the secondary symptoms of the illness, including depression and family dysfunction.  Also, family members help the social worker to understand the progress of the disease by providing additional information.  An appropriate framework must consider ethnicity as minority groups experience discrimination and decreased access to social services, putting them at increased risk. Interventions should address oppression, stereotyping, exploitation, and maintenance of culture vs. acculturation. |
| Muething, S. E., Kotagal, U. R., Schoettker, P. J., Del Rey, J. G., & DeWitt, T. G. (2007). Family-centered bedside rounds: A new approach to patient care and teaching. Pediatrics, 119(4), 829-832. doi:http://dx.doi.org/10.1542/peds.2006-2528 | Model Development | *Possibly:* Family-Centered Bedside Rounds | **Pediatric/Families:** This model targets families caring for children with acute conditions; the model was to be adapted to those caring for children with complex and chronic conditions  **Where:** Pediatrics, acute care hospital; model was developed and tested at Cincinnati Children’s Hospital  **HCP Involved:** A multidisciplinary team: attending physician, interns, residents, nurses and other involved ancillary staff; team members are assigned roles | **Objectives:**   - To improve communication between staff and family - To share decision-making, as family members are the experts on their child   **Desired Outcome/End Goals:** To decrease delays or complication at discharge, as the family is involved in setting goals and discharge planning  **Process/Intervention:** Teaching rounds take place either in the room or just outside in the hallway, with the family involved as active participants in updates in medical status, development of care plans, and discharge planning; both medical terms and lay language are used.   - The family decides how rounds are conducted, and whether or not they participate. - Introduction is a key component in ensuring families feel they are partners in the care-giving process. - Team efficiency is important – some members are given other tasks to complete during rounds such as orders, d/c summaries, prescriptions and home health care plans. - The intern or student assigned to the patient welcomes the family and clarifies the purpose of rounds. - At end of rounds, everyone is aware of and comfortable with treatment plan, therefore confusion is decreased and efficiency and effective communication are increased. - The teacher models appropriate family collaborative decision making for students and residents (i.e. Nodding, addressing fears or misunderstanding, asking questions, making eye contact, etc.) - Senior residents and teaching attendants ask families for permission to conduct additional teaching in the room. Parents usually like this and find it helpful. |
| Paone, M. C., Wigle, M., & Saewyc, E. (2006). The ON TRAC model for transitional care of adolescents. Progress in Transplantation, 16(4), 291-302. | Model Development | The ON TRAC model for transitional care of adolescents | **Adolescents/Pediatric:** This model targets transitional care of adolescents with chronic health conditions and their families, specifically applied to paediatric transplant.  **Where:** This model was developed at Children’s and Women’s Health Centre of British Columbia  **HCP Involved:** 14 Multidisciplinary teams of physicians, nurses, a social worker, dieticians, physiotherapists and occupational therapists | **Objectives:**   - To provide the tools and resources necessary for all adolescents to reach their attainable levels of independence, self-sufficiency, and self-worth while transferring safely and securely into adult healthcare services and adulthood.   Sub-objectives:   - To ensure all care provided to adolescents is family centered, youth focused, culturally sensitive, and developmentally appropriate. - To ensure that each individual leaving a pediatric program had been offered the skills and information to competently make safe healthcare decisions and access appropriate healthcare services.   **End Goal/Desired Outcome:** To encourage and support adolescences to prepare for the future.  **Process/Interventions:**   - ON TRAC is a 2-tiered service that provides education and information to:  1. Assist existing teams with transition issues and develop tools and resources that they can integrate into the provision of care 2. Support teams as they teach and empower adolescents and families  - On a regular basis beginning at 10 years of age, discuss issues with adolescents as guided by a clinical pathway framework that addresses self-advocacy; independent healthcare behaviours; sexual health; social supports; educational, vocational, and financial planning; and health and lifestyle - Clinical pathway/transition framework will get sent to the adult facility along with the complete medical record - The healthcare team must respect the need for peer acceptance and independence, and facilitate autonomous decision making and self-advocacy - Adolescents are given a health planner (“Your Plan-it”), the “Getting ON TRAC Workbook”, and “Cocktails”, a book listing information regarding interactions with medications and alcohol/drugs |
| Perrin, J. M., Romm, D., Bloom, S. R., Homer, C. J., Kuhlthau, K. A., Cooley, C., ... & Newacheck, P. (2007). A family-centered, community-based system of services for children and youth with special health care needs. *Archives of pediatrics & adolescent medicine*, *161*(10), 933-936. | Model Development; Literature Review | Family-centered community-based system of services for children and youth with special health care needs (CYSHCN) | **Pediatric:** Children and youth with special health care needs  **Where:** All levels of the community (defined as the available services, supports, and resources with which a family interacts given the child’s needs, the family’s economic resources and cultural background)  **HCP Involved:** physicians, school staff, social service providers, financial services, mental health providers, substance abuse providers, policy-makers | **Objectives:**   - To promote the healthy development and well-being of the child and family - To recognize and address the specific developmental needs of infants, children and adolescents, as well as important developmental transitions - To promote the cost-effective provision of services   **Process/Intervention:** Families are at the core of a spectrum of community-based services. Services must be accessible, flexible, responsive and targeted to address mental, physical, emotional and social needs. Families should be encompassed by spiritual resources, recreational programs, informal supports and services and voluntary organizations. Specific service elements involve, mental health, medical home/other, insurance/financing, transportation, juvenile justice, pubic safety, public health, housing, vocational services, education and social services.  When implementing such a system of services for CYSHCN, both the macro and micro levels of society must be considered and changed. The macro level includes agency constituents (federal, state and local) while the micro level includes community service systems (physicians, other health care providers, schools, public transportation, etc).  **Other Notes:** Although efforts have been made to address the needs of CYSHCN and their families, implementation of a well-integrated and comprehensive community-based system of services fully responsive to the needs of CYSHCN has not yet been fully realized. |
| Prelock, P. A., Beatson, J., Contompasis, S. H., & Bishop, K. K. (1999). A model for family-centered interdisciplinary practice in the community. Topics in Language Disorders, 19(3), 36-51. doi:http://dx.doi.org/10.1097/00011363-199905000-00005 | Model Development | Vermont Interdisciplinary Leadership Education for Health Professionals (VT-ILEHP) assessment and consultation model | **Pediatric:** Children with special health care needs and their families  **Where:** small towns and rural states (Vermont)  **HCP Involved:**  speech-language pathology, audiology, pediatrics, nursing, social work, nutrition, psychology, physical therapy, public administration, education | **Objectives:** (VT-ILEHP program as opposed to model objectives)   - To improve the health status of CSHCN and their families - To support training for health care professionals in the field - To prevent health risks - To coordinate health care - To foster community-based partnerships - To promote systems change through leadership   **Process/Intervention:**   1. Intake process : referral is made by special educator or pediatrician to the VT-ILEHP, a clinical coordinator conducts family and provider interviews to determine the perception of child’s needs, the community evaluation is developed by the family and adapted to their preferences, priorities and culture 2. Location of assessments: assessments are conducted at home and in the community (child care center, recreation activity, family’s home, school). Assessments consist of observations, interviews, and record review. 3. Information sharing: interdisciplinary reports, community follow-up meetings and resource notebooks |
| Prizant, B. M., Wetherby, A. M., Rubin, E., & Laurent, A. C. (2003). The SCERTS Model: a transactional, family-centered approach to enhancing communication and socioemotional abilities of children with autism and spectrum disorder...Social Communication, Emotional Regulation, and Transactional Support. *Infants & Young Children: An Interdisciplinary Journal of Early Childhood Intervention, 16*(4), 296-316. doi:https://dx.doi.org/10.1111/j.1744-6171.2006.00040.x | Model Development | The SCERTS Model, which prioritizes Social Communication, Emotional Regulation, and Transactional Support as the primary development of young children with ASD and their families | **Pediatric:** Young children with autism spectrum disorder (ASD) and their families  **Where:**N/A  **HCP involved:** (highlights multidisciplinary but unspecified)clinicians, educators, | **Objectives:** To address core challenges of children with ASD as they relate to social communication, emotional regulation, and transactional support.  **End Goal/Desired Outcome:** Improved functioning of children with ASD  **Process/Intervention:**   - Building a child’s capacity to initiate communication with a conventional, symbolic system - Develop self- and mutual-regulatory capacities to regulate attention, arousal, and emotional state - Provide guidance and support to all who interact with a child with ASD on a regular basis since they are potential developmental facilitators - Use of communicative partners - Established goals require well-designed, semistructured activities, and more varied social contexts - Identify the types of visual and organizational supports that may be helpful based on developmental capacities and needs, also relative to the social contexts in the child’s life - Support families in reference to educational and emotional supports - By helping parents to think clearly about their priorities, develop appropriate expectations and realistic, achievable goals for their child’s development - Parents are respected as having ultimate “ownership” of the decisions that are made for child and family - Professionals must “keep the hope alive” by emphasizing child’s strengths, needs, positive development and change, and “next steps”   **Other Notes:** The following features are revealed as essential for comprehensive educational/treatment model:   1. Model is based on most current research in child development and ASD 2. Flexible enough to incorporate different perspectives (i.e. development and contemporary applied behavior analysis – ABA) 3. Can be applied in an individualized manner while addressing core deficits of ASD 4. Is family-centered, taking into account individual differences across families, their priorities, their involvement in decision making |
| Raina, P., O'Donnell, M., Rosenbaum, P., Brehaut, J., Walter, S. D., Russell, D., . . . Wood, E. (2005). The health and well-being of caregivers of children with cerebral palsy. Pediatrics, 115(6), e626-636. doi:https://dx.doi.org/10.1542/peds.2004-1689 | Model Development; applied structural equation modeling with data from a large cohort of caregivers of children with CP |  | **Pediatric:** Primary caregiver of children with cerebral palsy  **Where:**  **HCP involved:** | **Objectives:** To examine the direct and indirect associations between caregiver characteristics, sources of caregiver stressors, family functioning, and informal social support on the well-being of the caregivers of children with cerebral palsy.  Model objectives:  See Raina et al, 2004.  **End Goal/ Desired Outcome:** Better support caregivers of children with cerebral palsy.  **Process/Intervention:**  Standardized, self completed parental questionnaires  Face-to-face home interviews  Instrument**s:**  *Child*  Motor severity: GMFCS (Gross Motor Function in Children with Cerebral Palsy Scale)  Activities of daily living: Pediatric Evaluation of Disability Inventory, Part 1  Cognitive function: Health Utilities Index, selected questions  Child behavior: Survey Diagnostic Instrument (SDI)  *Caregiver*  Caregiver health status: Medical Outcomes Study, Short Form 36 Health Survey (SF36)  Perception of formal care: Measures of Processes of Care  Stress Management: Coping Health Inventory for Parents  SES of caregivers, chronic health conditions: National Longitudinal Study of Children and Youth (NLSCY)  Distress, depression, mastery, self-esteem: National Population Health Survey (NPHS)  Health status: McMaster Health Unity Index (HUI), in NPHS  Caregiving assistance: Pediatric Evaluation of Disability Inventory (PEDI): Parts II, III  Job-caregiving conflict informal social support: Pearlin’s Scale, Social Network and Frequency of Contact Index in NPHS, Social Provision Scale (SPS) in NLSCY  Family functioning: Family Assessment Device (FAD) in NLSCY  **Analyses:**  Structural equation modeling with log linear modeling (LLM) as an adjunct  Model fit: covariance matrices and maximum-likelihood estimation method, Root-mean-squared error of approximation (RMSEA), Nonformed Fit Index (NNFI), Comparative Fit Index (CFI)  **Other Notes:**   - Most important predictors of caregivers’ well-being were child behavior, caregiving demands, and family functioning. - Higher level of child behavior problems was associated with lower levels of caregiver psychological and physical health. Moreover, fewer child behavior problems were associated with higher caregiver self-perception and increased ability to manage stress. - Decreased caregiving demands were associated with better with better physical and psychological well-being. - Higher family functioning was associated with better caregiver psychological and physical health. - Caregivers’ self-perception and stress management were significant predictors of caregivers’ psychological health but not of their physical well-being. - Caregivers’ higher self-esteem, sense of mastery over the caregiving situation, and more use of stress management strategies predicted better psychological health. - Gross income and social support indirectly affected psychological health outcome. - Self-perception, stress management, gross income, and social support indirectly affected physical health outcomes. |
| Regan, K. M., Curtin, C., & Vorderer, L. (2006). Paradigm shifts in inpatient psychiatric care of children: approaching child- and family-centered care. *Journal of Child & Adolescent Psychiatric Nursing, 19*(1), 29-40. | Model Development; Literature review | Child- and Family-Centered Care (CFCC) Model | **Pediatric:** children in inpatient psychiatric care and their families.  **Where:** Inpatient child psychiatric unit in Cambridge, Massachusetts  **HCP involved:** nurses | **Objectives:** To create a new culture on the unit that emphasized collaboration rather than adversial relationships.  **Prcoess/Intervention:**   - Values: Nurturance, opportunities to learn and teach, and the provision of choices based on individual needs. - The adoption of a collaborative problem solving (CPS) model in responding to children’s behavioural difficulties: instead of rewards and consequences, staff try to understand the child’s behaviour, prevent meltdowns, maintain the child’s trust and resolve the problem in a mutually satisfactory way - Open hours for parents: parents are therefore seen as partners in caring for the child. Open hours allow staff to interact more with parents, educating them on ways to approach behavioral difficulties using the CPS model. - Trauma-sensitive protocols and procedures: Children are often on the unit because of traumatic experience(s). Building trusting relationships through a nurturing approach. Hugging, patted, affectionately approached, jokes are all appropriate ways to show this. Bedtime and medical procedures are approached with sensitivity. Decrease of restraints.   **Other Notes**: The traditional model of psychiatric care for children, which emphasized group norms, consistency and control, was questioned due to increased use of mechanical restraints and locked-door seclusion, staff dissatisfaction, and general negativity on the unit. |
| Reid Ponte, P., & Peterson, K. (2008). A patient- and family-centered care model paves the way for a culture of quality and safety. *Critical Care Nursing Clinics of North America, 20*(4), 451-464. doi:https://dx.doi.org/10.1016/j.ccell.2008.08.001 | Model Development | Interdisciplinary leadership and governance model | **Adult:** oncology patients and female patients (and their families)  **Where:** Dana Farber Cancer Institute (DFCI) and Brigham Women’s Hospital  **HCP Involved:** nurses, physicians, administrators, other disciplines critical to oncology care (e.g. pharmacists, social workers, nutritionists, other allied health staff) | **Objectives:** To collaborate and partner with patients, families, and with one another  **Desired Outcome**: Improved patient quality and safety  **Process/Intervention:**   1. Creating 2 Patient and Family Advisory Councils (1 for pediatrics, 1 for adult care) 2. Key values, structures, and processes: 3. Interdisciplinary leadership through a nurse-physician-administrative leadership model 4. Patient and family involvement in organizational decision making 5. Transparency and openness by executive leadership 6. A fair and just culture and systems for error identification and mitigation 7. Respectful interdisciplinary collaboration and team effectiveness 8. Magnet program of nursing excellence (blueprint for achieving excellence in nursing practice and patient care) 9. Evidence-based practice, clinical effectiveness, and continuous learning 10. Systems thinking, process improvement, and clinical quality improvement framework   **Other Notes:** Concerns were raised by patients about the impact on patient care once the Dana Farber Cancer Institute (DFCI) entered into a joint clinical partnership with the Brigham and Women’s Hospital (BWH). DFCI’s interdisciplinary executive leadership team questioned how they could assure the needs of patients and families would drive decision making while being at the center of care. Additionally, following two medical errors, of which one was fatal, the DFCI recognized that at the time the patient’s concerns were not considered as useful in guiding clinical care. Witnessing the consequences of these system-level flaws was a wake-up call for the need to commit to patient- and family centered care, and patient safety and quality. |
| Romero-Daza, N., Ruth, A., Denis-Luque, M., & Luque, J. S. (2009). An alternative model for the provision of services to HIV-positive orphans in Haiti. *Journal of Health Care for the Poor and Underserved, 20*(4 SUPPL.), 36-40. | Model Development; Case Study | Family-oriented model of care | **Pediatric:** HIV-positive Haitian orphans  **Where:** a rented house situated in a lower middle-class neighborhood  *HCP Involved:* physicians, nutritionists, psychologist, social worker, nurse | **Objectives:**   1. Providing services to a small group of orphans in a family-oriented setting 2. Employing HIV-positive women and integrating children into the community 3. Providing a balanced diet with supplementation and access to safe water 4. Ensuring access to formal education 5. Ensuring health care   **Desired Outcome:** Improve the care of HIV-positive orphans by restoring a sense of family and individuality for each child.  **Process/Intervention**   1. Ensuring that the number of children served remains small in order to provide individual attention and family-centered care. 2. Employing HIV-positive women as caretakers providing motherly care for the children. They live in a house that is unmarked to avoid stigma and normalize the experience as much as possible. 3. Doctor and nutritionists that care for the children and caretakers give recommendations for proper nutritional supplements; municipal water delivery ensured with supplies required to boil it 4. Housing located near local schools, tuition, supplies, and uniforms provided 5. Free routine care through local hospital (monthly checkups and ART medication) for children and caregivers; visiting nurse between checkups   **Other Notes:** Due to budget constraints, these orphanages provide only basic services (food, shelter, medical care); personalized care is not possible. The needs of AIDS orphans who are also HIV-positive are not met. |
| Sharifah, W. W., Nur, H. H., Ruzita, A. T., Roslee, R., & Reilly, J. J. (2011). The Malaysian Childhood Obesity Treatment Trial (MASCOT). Malaysian Journal of Nutrition, 17(2), 229-236. | Model Development; RCT | Malaysian Childhood Obesity Treatment Trial (MASCOT) Treatment Program | **Pediatric:** obese children of primary school age (7 to 11 years old)  **Where:**  Malaysia  **HCP Involved:** dietician/nutritionist, psychologist | **Objectives:**   1. assist the parent and child in raising their awareness of their lifestyle 2. help them focus on the aspects of their lifestyle which require changes 3. motivate the child and family to make lifestyle changes 4. help the child and family monitor those changes   **Intervention/Process:** eight-session intervention (with a total dose of patient contact of around  eight hours) delivered by a dietitian/nutritionist to groups of parents   - 1. Wake up call : benefits and sacrifice of weight management, readiness to change   2. Eat well, be well” energy balance, healthy eating, goal setting, self-monitoring   3. Be active!: motivate child to initiate physical activity, decrease sedentary behaviour, goal setting, self-monitoring   4. Make a better life: daily breakfast, family meal, fast food, label reading, problem-solving, self-monitoring   5. I feel good: parenting skills, being a good role model, dealing with stress, problem solving, self-monitoring   6. Let’s cook together: making foods together, modifying food in a healthy way   7. Simply the best: understand relapse, improve current diet and physical activity, tips for maintaining successful routine, problem-solving   8. Sharing is caring: sharing tips, long-term goal setting   **Other Notes:** Development of the treatment programme was based on an adaptation of guidelines to the Malaysia setting. The MASCOT treatment focuses on change in three key behaviours recommended as the principal targets of obesity treatment in recent systematic reviews of childhood obesity treatment (Luttikhuis et al., 2009) and evidence based clinical guidelines on childhood obesity treatment (NICE, 2006; ADA, 2006) such as reductions in sedentary behaviour, particularly screen-time, increases in physical activity and changes in diet. Parents are targeted as the main agents of lifestyle change, as recommended by recent systematic reviews and clinical guidelines. |
|  |  |  |  |  |
| Sisterhen, L. L., Blaszak, R. T., Woods, M. B., & Smith, C. E. (2007). Defining family-centered rounds. *Teaching & Learning in Medicine, 19*(3), 319-322. doi:https://dx.doi.org/10.1080/10401330701366812 | Model Development | Family-centred Rounds | **Adult:** This model is targeted toward patients in hospital and their families.  **Where:** This model can be implemented in any in-patient ward in academic hospitals.  **HCP Involved:** Physicians and medical residents | **Objectives:**   1. To improve patient and family satisfaction by encouraging their active participation in rounds’ discussions 2. To improve resource utilization 3. To create a “patient-centered” environment 4. To improve staff job satisfaction 5. To improve outcomes 6. To model to medical students: effective patient communication, clinical ethics, and professionalism   **Desired Outcome:** Improved patient and family satisfaction  **Process/Intervention:** Family-centered rounds are defined as interdisciplinary work rounds at the bedside in which the patient and family share in the control of the management plan as well as in the evaluation of the process itself. Families and patients must give permission to these rounds. Attention is paid to ask for family and patient feedback, as well as to use language that is understandable.  **Other Notes:** Family-centered rounds were developed partly in response to physician’s disappointment in decreasing bedside rounds in favor of conference table rounds. Family-centered bedside rounds were developed to increase communication and coordination of services, provide efficiency in developing a unified care plan, and to model the family-centered approach to residents. |
| Tluczek, A., Zaleski, C., Stachiw-Hietpas, D., Modaff, P., Adamski, C. R., Nelson, M. R., . . . Josephson, K. D. (2011). A tailored approach to family-centered genetic counseling for cystic fibrosis newborn screening: the Wisconsin model. Journal of Genetic Counseling, 20(2), 115-128. doi:https://dx.doi.org/10.1007/s10897-010-9332-y | Model Development; Literature Review | Wisconsin Model | **Parents of Peds:** Parents of infants identified as CF carriers  **Where:** N/A  **HCP involved:** genetic counselor (CF expert) | **Objectives:** To   1. minimize parents’ distress 2. facilitate parents’ understanding 3. increase parents’ capacities to use genetic information 4. enhance parents’ experiences with genetic counseling   **Intervention/Process**  To achieve these outcomes, counselors engage in an iterative process of evaluating parents’ needs and tailoring responses accordingly. Corresponding assessment and intervention domains include the therapeutic environment, parents’ and infants’ emotional needs, parents’ informational needs, and the development of a follow-up plan.  When parents attend genetic testing counseling session:   1. Creating a Therapeutic Environment: counselor attends to family’s physical comfort, allows enough space in a private room (confidentiality), communicate genuine interest in parents well-being, and infuse cultural sensitivity in their interactions 2. Addressing Parents’ Emotional Needs: Using parents’ subjective reports and the counselor’s objective observations, counselors form hypotheses about parents’ emotional states and then test these hypotheses by explicitly asking parents about them. Emotion-focused interventions include reflecting parents’ feelings, redirecting the conversation to address the source of their concerns, normalizing parents’ reactions, pausing to allow time for them to regain their composure, and instilling realistic hope. 3. Addressing Parents’ Informational Needs: the type and amount of information is titrated to individual parent requests. Counselors match the level of technical description in verbal explanations to the parents’ baseline knowledge and explicit request for details 4. Developing a Follow-Up Plan: A follow-up plan is collaboratively developed with parents to provide them access to additional resources as needed. The plan includes mailing parents a letter summarizing the session, the counselor’s contact information, and who to contact if additional questions or concerns arise.   **Other Notes**: Research findings repeatedly document knowledge deficits, emotional distress, and lingering worry about infant health among parents of infants identified as CF carriers. The mandatory nature of NBS combined with the vulnerable emotional states of parents resulting from the receipt of unsolicited, unexpected abnormal results present unique challenges that may merit a more psychosocially oriented approach to genetic counseling than typically required. |
| Tyler, D. O., & Horner, S. D. (2008). Family-centered collaborative negotiation: a model for facilitating behavior change in primary care. Journal of the American Academy of Nurse Practitioners, 20(4), 194-203. doi:https://dx.doi.org/10.1111/j.1745-7599.2007.00298.x | Model Development; Literature Review | Collaborative Negotiation Model | **Who:** This model applies a family-centred approach to overweight children and their families  **Where:** The authors recommend that this model be implemented in primary care settings.  **HCP Involved:** Primary care providers | **Objectives:**   1. To promote child health through active family participation 2. To support family-child relationships 3. To enhance family motivation and the abilities to change behaviour   **Process/Intervention:** This model combines a family-centred interaction approach called Touchpoints (Brazelton, 1992) with brief negotiation strategies to address health risks in children. By using a non-confrontational and family-centred approach, caring and collaborative relationships between families and clinicians can be fostered which helps to minimize resistance to health promotion facilitation. Behaviour specialists and other supports are also used.  The model contains three constructs:   1. Factors that affect the child’s health and risk status (i.e. Genetics, family environment, knowledge, attitudes, etc.) 2. A family-centred intervention with the PCP to negotiate behaviour change (PCP collaborates with parent and child) 3. Health indicators that reflect outcomes of PCP-family interaction (i.e. Body mass index, activity level, eating behaviours, etc.)   Touchpoints is a strength-based (opposed to deficit-based), empathic model of care where practitioners collaborate with parents to anticipate developmental and situational transitions. It assumes that parents have strengths and want the best for their child(ren).  Brief negotiation is an efficient form of motivational interviewing used to increase clients’ motivation and readiness to change. There are four fundamental tasks of brief negotiation strategies:   1. Setting a mutually agreeable agenda 2. Making shared decisions and setting targets 3. Assessing and strengthening motivation and confidence regarding the planned actions 4. Exchanging information   **Other Notes:** see p. 195 for model  Real lifestyle change is most likely to occur when a family-centred approach is used because “family is the context where health is learned, lived and experienced”.   - Primary care providers need a framework to adequately address problems and build skills in family-centred intervention. - This model was developed because many primary care providers feel uncomfortable addressing psychosocial or lifestyle issues, such as behaviours that contribute to overweight children. |
| Vraniak, D. (1997). Mapping contexts for supporting American Indian families of youth with disabilities. Families, Systems and Health, 15(3), 283-302. | Model Development | The Meendendiwin Model (Ojibwa for “mutual gifting”) | **Pediatric:** This model targets American Indian families who have youth with disabilities.  **Where:** Areas of the USA where American Indian communities exist.  **HCP Involved:** The entire system of support and services | **Objectives:**   1. To move away from a hierarchical, control-oriented system toward a horizontal, affiliative and ecological system: from “one serving many” to “many serving one” 2. To move away from a segmental system to a more coherent, multidisciplinary framework 3. To link family support and community support through an integrated approach   **Desired Outcome/End Goal:** To strengthen families’ capabilities and to help families realize that they are responsible for its youth  **Process/Intervention**   - Comprehensive assessment under one roof: family-driven and family-defined multidisciplinary assessment to create a comprehensive treatment plan - Family care coordination: A facilitator or team helps families navigate the available resources - Integrated caring program: A prevention intervention program with integrated school-based, home-based, and community-based components - Cultural community value-based: This new system must work within traditional cultural forms to foster trusting relationships   **Other Notes:** This model reconfigures and transforms mainstream medical, educational, mental health and social services into a form that most appropriately meets the multiplicity and severity of the needs of this population. |
| Ward, K. G. (1999). A TEAM approach to NICU care. RN, 62(2), 47-49. | Model Development | The TEAM model (teach, empower, assess, and monitor) | **Pediatrics:** critically ill infants and their parents  **Where:** NICU  **HCP Involved**: Nurses | **Objectives:** To make the infant’s stay less upsetting, promote bonding between parents and child, and help improve postpartum care.  **Desired Outcome/End Goal**: Improve the NICU experience  **Process/Intervention:** Integrate the parents in the infant’s daily treatment plan by providing routine, hands-on care, providing continuous information on the infant’s condition, and providing practical and psychosocial support to ease the transition from hospital to home.   1. Teach: By using language that is familiar to the family, prepare them for the NICU experience. Explain treatment plans, medications, and equipment. Encourage parents to touch, talk, and hold the infant (condition permitting). Ensure that they understand the unit’s rules and policies. 2. Empower: Provide the family with the tools necessary so that they can take over the basic care of the infant, and be confident about it. Make sure that they know how to operate the equipment that they will be using at home. 3. Assess: Evaluate the family’s needs and make referrals to specialists when necessary (breastfeeding, social work, etc). Provide emotional support when needed. Provide them with contact information to support groups and other local groups. 4. Monitor: Analyze how well the family-centered model is meeting the needs of the parents. |
